# Transcriptome analyses reveal molecular mechanisms underlying phenotypic differences among transcriptional subtypes of glioblastoma

**DOI:** 10.1111/jcmm.14976

**Published:** 2020-02-24

**Authors:** Yuan‐Bo Pan, Siqi Wang, Biao Yang, Zhenqi Jiang, Cameron Lenahan, Jianhua Wang, Jianmin Zhang, Anwen Shao

**Affiliations:** ^1^ Department of Neurosurgery Second Affiliated Hospital School of Medicine Zhejiang University Hangzhou China; ^2^ Department of Radiology The Affiliated Hospital of Medical School of Ningbo University Ningbo University School of Medicine Ningbo China; ^3^ Department of Nuclear Medicine Sir Run Run Shaw Hospital Medical College of Zhejiang University Hangzhou China; ^4^ Department of Neurosurgery Shanghai Ninth People's Hospital Shanghai Jiaotong University School of Medicine Shanghai China; ^5^ Cixi Institute of Biomedical Engineering Ningbo Institute of Materials Technology and Engineering Chinese Academy of Sciences Ningbo China; ^6^ University of Chinese Academy of Sciences Beijing China; ^7^ Burrell College of Osteopathic Medicine Las Cruces NM USA; ^8^ Center for Neuroscience Research School of Medicine Loma Linda University Loma Linda CA USA; ^9^ Brain Research Institute Zhejiang University Hangzhou China; ^10^ Collaborative Innovation Center for Brain Science Zhejiang University Hangzhou China

**Keywords:** angiogenesis, ER stress, GBM, molecular subtypes, transcriptome, WGCNA

## Abstract

Using molecular signatures, previous studies have defined glioblastoma (GBM) subtypes with different phenotypes, such as the proneural (PN), neural (NL), mesenchymal (MES) and classical (CL) subtypes. However, the gene programmes underlying the phenotypes of these subtypes were less known. We applied weighted gene co‐expression network analysis to establish gene modules corresponding to various subtypes. RNA‐seq and immunohistochemical data were used to validate the expression of identified genes. We identified seven molecular subtype‐specific modules and several candidate signature genes for different subtypes. Next, we revealed, for the first time, that radioresistant/chemoresistant gene signatures exist only in the PN subtype, as described by Verhaak et al, but do not exist in the PN subtype described by Phillips et al PN subtype. Moreover, we revealed that the tumour cells in the MES subtype GBMs are under ER stress and that angiogenesis and the immune inflammatory response are both significantly elevated in this subtype. The molecular basis of these biological processes was also uncovered. Genes associated with alternative RNA splicing are up‐regulated in the CL subtype GBMs, and genes pertaining to energy synthesis are elevated in the NL subtype GBMs. In addition, we identified several survival‐associated genes that positively correlated with glioma grades. The identified intrinsic characteristics of different GBM subtypes can offer a potential clue to the pathogenesis and possible therapeutic targets for various subtypes.

## 1 INTRODUCTION

GBMs are the most common malignant primary brain tumour found in adults. The primary treatment for GBM involves maximal surgical resection, followed by radiotherapy and alkylating chemotherapy with temozolomide.^1^, ^2^ The median survival of optimally treated GBM patients is 14 months, with a 26% 2‐year survival rate.^1^ Although the overall prognosis is poor, some patients may have significantly better responses to therapy and outcome.^3^ In order to categorize the tumours, Phillips et al identified three distinct subtypes of high‐grade astrocytomas by performing gene expression profiling. These subtypes are described as: proneural (PN), mesenchymal (MES) and proliferative (Prolif).^4^ Subsequently, Verhaak et al performed an unsupervised transcriptome clustering of 1740 expressed genes derived from 202 GBMs, and identified four clusters: proneural (PN), neural (NL), mesenchymal (MES) and classical (CL),^4^ which were closely correlated with genomic abnormalities.

Each subtype has its own phenotypes, and the PN and MES subtypes have been described most consistently. Patients with the PN subtype GBM are younger, and this subtype is characterized by platelet‐derived growth factor receptor, alpha polypeptide (PDGFRA) amplification and isocitrate dehydrogenase 1(IDH1) mutation. Patients with the PN subtype GBM have an improved prognosis when compared with the other subtypes, which correlates with the increased IDH1 mutation rate found in this subtype. However, the PN subtype is unresponsive to chemical and radiation therapy. The MES subtype of GBM overexpresses the mesenchymal marker, which is associated with the deletion and silencing of the chromosomal tumour suppressor gene, NF1, and the point mutation of the phosphatase and tensin homolog (PTEN). Moreover, the MES subtype is associated with poorer survival and a higher proportion of necrosis. However, the biological processes and corresponding gene programmes underlying these phenotypes were not well known. A thorough analysis of the biological processes and molecular mechanisms of these phenotypes would help us understand the intrinsic characteristics of different subtypes, which can offer insight into the pathogenesis and possible therapeutic targets for each subtype. Moreover, the lack of a powerful bioinformatics analysis method prevented previous studies from thoroughly exploring the biological processes and molecular mechanisms behind these distinct phenotypes.^5^ However, a powerful systematic analysis method, weighted gene co‐expression network analysis (WGCNA), allows the identification of different gene networks based on co‐expression relationships between all expressed genes across samples.^6^, ^7^ Genes in the same network, or module, often execute similar biological functions, including neural system development, ER stress response, cell cycle, inflammatory response and angiogenesis. In addition, the networks that function within specific molecular subtypes of GBMs were also identified based on module‐trait correlations, allowing us to identify the biological processes and molecular programmes behind specific phenotypes of different subtypes. Furthermore, WGCNA allows us to select more central genes with higher intramodular connectivity within a single module, thereby removing confounding factors and producing convincing results. WGCNA, when based on transcriptome data, can dissect various ‘ingredients’ (ie different pathological events) in the ‘soup’ of the GBMs.^8^

In this study, we used WGCNA to identify several gene modules associated with four different molecular subtypes, which are involved in major biological processes. Firstly, we identified some candidate signature genes for various molecular subtypes. Secondly, we revealed that the radioresistant/chemoresistant gene signatures only exist in the Verhaak et al PN subtype, but not in the PN subtype that was described by Phillips et al. Thirdly, we revealed that the tumour cells in the MES subtype are under ER stress and that angiogenesis and the immune inflammatory response are both significantly elevated in this subtype. The molecular mechanisms of the ER stress response, immune inflammatory response and angiogenesis were uncovered. Next, we revealed for the first time that genes associated with alternative RNA splicing are significantly up‐regulated in the CL subtype GBMs and that genes pertaining to energy synthesis are markedly elevated in the NL subtype GBMs. Lastly, we identified several survival‐associated genes that were positively correlated with glioma grades, using the RNA‐seq and immunohistochemical data.

## 2 MATERIALS AND METHODS

### 2.1 Data set acquisition and pre‐processing

RNA sequencing data (count files) and the corresponding clinical information of GBM samples were downloaded from the data portal of The Cancer Genome Atlas (TCGA, https://cancergenome.nih.gov/). The count files were normalized through the DESeq package.^9^ The samples that lacked any subtyping traits were eliminated. Gene Expression Omnibus (GEO, https://ncbi.nlm.mih.gov/geo/) is a public functional genomics data repository, which supports various types of high‐throughput experimental data submission. The GEO series (GSE4271 and GSE16011) contain the raw data and clinical information of samples. Raw gene expression microarray data (CEL files) of high‐grade gliomas were downloaded from the http://www.ncbi.nlm.nih.gov/geo/query/acc.cgi?acc=GSE4271. The raw probe‐level data in CEL file were processed using the robust multi‐array average (RMA) algorithm in the Affy package^10^ of the R language, including background correction, quartile data normalization and conversion into expression measures. For genes that correspond to multiple probes, we used the average probe value as the expression value. Missing data in gene expression matrixes were imputed with the k‐nearest neighbour (KNN) approach (*k* = 10).^11^

### 2.2 Construction of co‐expression networks

The weighted gene co‐expression network was constructed for the GBM data set to identify gene modules associated with expression patterns of different molecular subtypes of GBMs following a previously described algorithm.^6^ The co‐expression network was constructed by the R package WGCNA.^7^ In brief, the correlation network was first constructed by creating a matrix of Pearson's correlation between all pairwise genes. Then, the obtained correlation matrix was transformed into a weighted adjacency matrix, using a power (β) of 4.^6^ Next, the topological overlap matrix (TOM), a measure of network interconnectedness, was calculated.^6^ On the basis of the TOM‐based dissimilarity measure, we used average linkage hierarchical clustering to classify genes with similar expression patterns into the same modules with a minimum size of 30.^7^ Modules were determined by using a dynamic tree‐cutting algorithm.^6^ Using a merging threshold function at 0.25, the final modules were identified.^6^ Rather than using all 20 456 annotated genes to describe the molecular events in GBMs, we instead selected 25% of the genes that had the greatest variance. A total of 5114 genes were input into WGCNA. A total of 148 GBMs were clustered via the expression of the 5114 genes, and 3 GBMs were excluded as outliers. In this study, we identified 19 modules for the GBM data set.

### 2.3 Calculating module eigengene, module membership and intramodular connectivity

The principal component analysis of each module obtained the first principal component, which was regarded as module eigengene (ME) in WGCNA. The expression pattern of each module in various subtypes of GBMs was summarized by the corresponding module eigengene. Each gene's module membership (MM) for a given module was then estimated as the Pearson correlation between that gene and the ME. The gene with a higher MM plays a more important role in the corresponding module; thus, genes with an MM > 0.6, defined as central genes, were selected for subsequent analyses. The intramodular connectivity of one gene was defined as the sum of the correlation coefficients with other nodes in the corresponding module. The genes with higher intramodular connectivity also play decisive roles in the corresponding module.

### 2.4 Identification of Clinically significant modules and Hub genes

**Figure 1:**
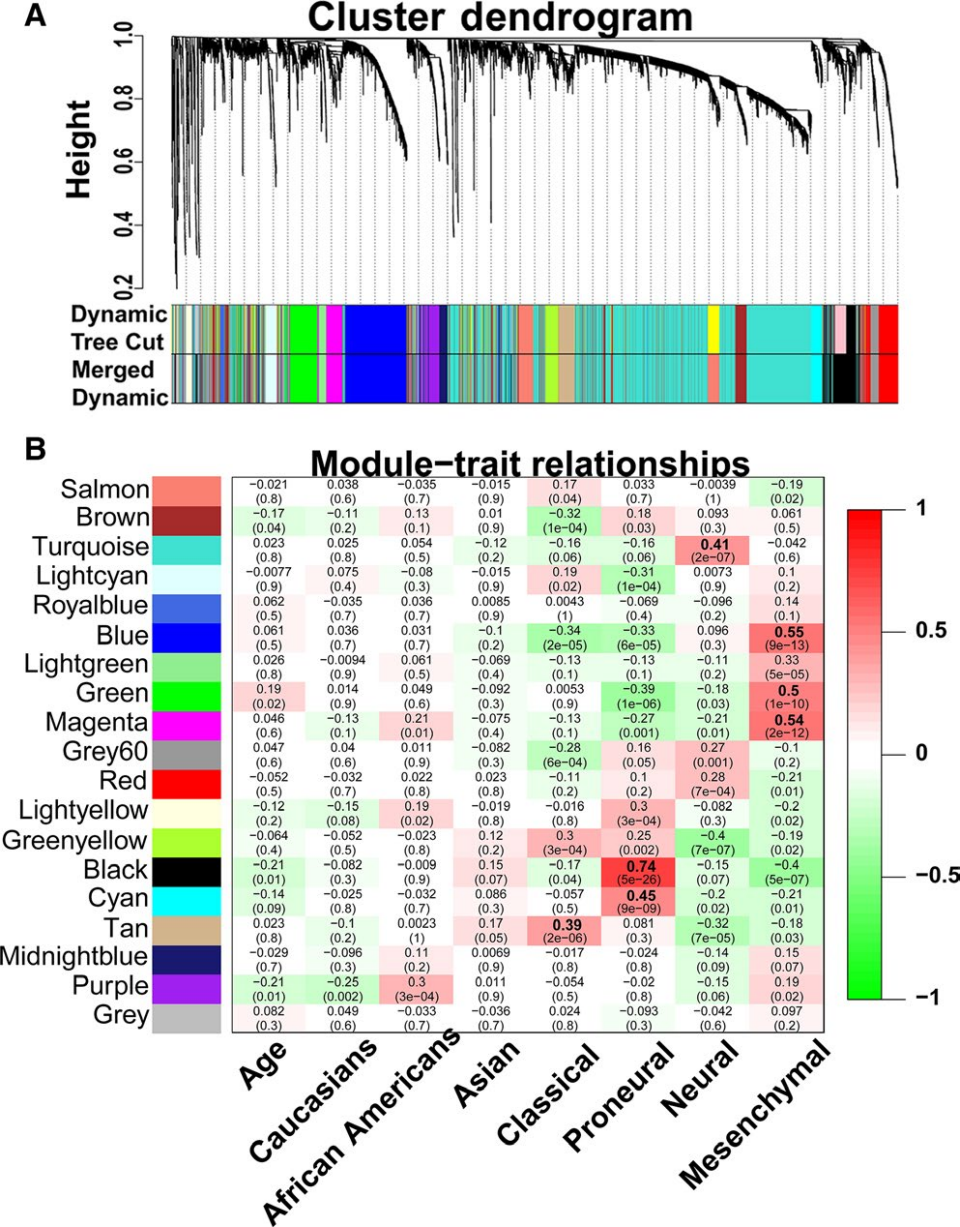
Weight gene co‐expression network analysis (WGCNA) identified molecular subtype–specific modules. (A) Gene dendrogram for the GBM data set is shown. (B) Heatmap of module‐trait relationships

The Pearson correlation between MEs and clinical traits was calculated to identify the most significant modules. The correlation coefficients and corresponding *P*‐values are shown in Figure 1B. The top 30 genes with the highest intramodular connectivity within each module were referred to as intramodular hub genes.

### 2.5 Gene ontology analysis

Gene ontology (GO) enrichment analysis was performed with the DAVID platform (DAVID 6.8, https://david.ncifcrf.gov/).^12^ Genes with MM > 0.6 within each module were utilized during GO enrichment analyses. GO terms with the Benjamini‐corrected *P*‐values < 0.05 are shown in Table S3. There are three categories in GO analysis: biological process (BP), cellular component (CC) and molecular function (MF).

### 2.6 Visualization of key networks

All connections of genes with MM > 0.6 in each module were selected, and Cytoscape 3.6.0 was used to visualize the top 250 connections (based on topological overlap). The triangular and square nodes represented known signature genes of certain molecular subtypes from Verhaak et al^5^ and Phillips et al,^4^ respectively. The diamond‐shaped nodes represented the overlapping signature genes from both studies. The hub genes in each module were marked as large‐sized nodes. The red nodes were identified as the candidate signature genes that we predicted in our study.

### 2.7 Gene set enrichment analysis (GSEA)

The gene expression profiles of GBMs in TCGA data set were analysed by GSEA (http://software.broadinstitute.org/gsea/index.jsp).^13^ According to expression levels of hub genes, 145 GBMs were divided into two groups, with the criteria that the expression level was above or below the median. The chemical and genetic perturbations (CGP) of curated gene sets (C2), BP, CC and MF in the Molecular Signatures Database (MSigDB, version 6.2), were analysed as a gene set database. The number of permutations was 1000, and the other parameters were set by their default values. The gene sets with a nominal *P*‐value < 0.05 were considered statistically significant.

### 2.8 Accessing immunohistochemistry data

The Immunohistochemistry results were acquired from the Human Protein Atlas (HPA, https://www.proteinatlas.org/) database.^14^ The immunohistochemistry results from the HPA database were used to compare the protein levels of selected genes among normal brain tissues, low‐grade gliomas (LGGs) and high‐grade gliomas (HGGs).

### 2.9 Statistical analysis

SPSS software (version 22.0, SPSS, Chicago, IL, USA), GraphPad Prism (version 6.0, GraphPad Software, San Diego, CA, USA) and R language (3.4.0) were used for statistical analysis. The results are presented as mean ± SEM. Statistical differences were determined by Student's *t* test for two‐group comparisons, or ANOVA followed by Tukey's test for multiple comparisons among more than two groups. Survival analysis was performed with the Kaplan‐Meier method. The statistical significance of differences between the two groups was evaluated using the log‐rank test. *P*‐value < 0.05 was considered a statistically significant difference. Bar plots, scatter plots and Kaplan‐Meier survival plots were generated using GraphPad Prism. Other plots, including dendrograms and heatmaps, were produced by R language (3.4.0).

## 3 RESULTS

### 3.1 Constructing the GBM co‐expression network

Identification of genes with expression levels that are highly correlated may shed light on biological and pathological events occurring in GBM and candidate signature genes. A total of 145 GBM samples were analysed in this study. To better describe molecular events in GBMs, a quarter of all 20 456 genes with the greatest variance, including 5114 genes, were selected for analysis. Based on the expression of 5114 genes, hierarchical clustering of 145 GBM samples was performed (Figure S1A). The average age of these samples was 60.32 years. The samples included 36 (24.8%) PN, 38 (26.2%) CL, 46 (31.7%) MES and 25 (17.2%) NL GBMs. In addition, of the samples obtained, 10 (6.9%) samples came from African Americans, 5 (3.4%) from Asians and 130 (89.7%) from Caucasians (Figure S1A). Next, we performed weighted gene co‐expression network analysis (WGCNA)^6^, ^7^ to identify the molecular subtype related to the network in GBMs. The power (β) of 4 was selected as a soft‐thresholding parameter to ensure a scale‐free network (scale *R* ^2^ = 0.91) (Figure S1B,C). WGCNA identified 19 modules (designated as 19 different colours) of co‐expressed genes (Figure 1A).

### 3.2 Identifying molecular subtype‐related modules

To identify the module that is significantly associated with the clinical traits, we performed a Pearson correlation between the module eigengenes and the clinical traits. The results of the module‐trait relationship were visualized as a heatmap with correlation coefficients and p‐values (Figure 1B). Based on the significant module‐trait relationships, we identified 7 modules strongly associated with different subtypes. Furthermore, based on the eigengene expression in each module and the traits of four molecular subtypes, hierarchical clustering was used to quantify module similarity and to determine the correlation between subtypes and specific modules (Figure S1D). A detailed eigengene adjacency of the traits and all of the modules are shown as a heatmap in Figure S1E. In addition, the expression of genes across the samples in these modules was visualized as heatmaps and eigengenes, also shown as bar plots, which further indicated that eigengene expression of different modules was significantly higher in corresponding subtypes of GBMs (Figure S2). The correlations of the module membership (MM) and gene significance (GS) are depicted as a scatter plot in Figure S3A–G, which indicated that the genes that significantly correlated to a specific subtype of GBM also occupied the central part of the corresponding module. Taken together, the modules highly correlated with four subtypes (black, cyan, blue, green, magenta, tan and turquoise modules) were selected for the following analyses.

### 3.3 Identifying candidate PN signature genes

**Figure 2:**
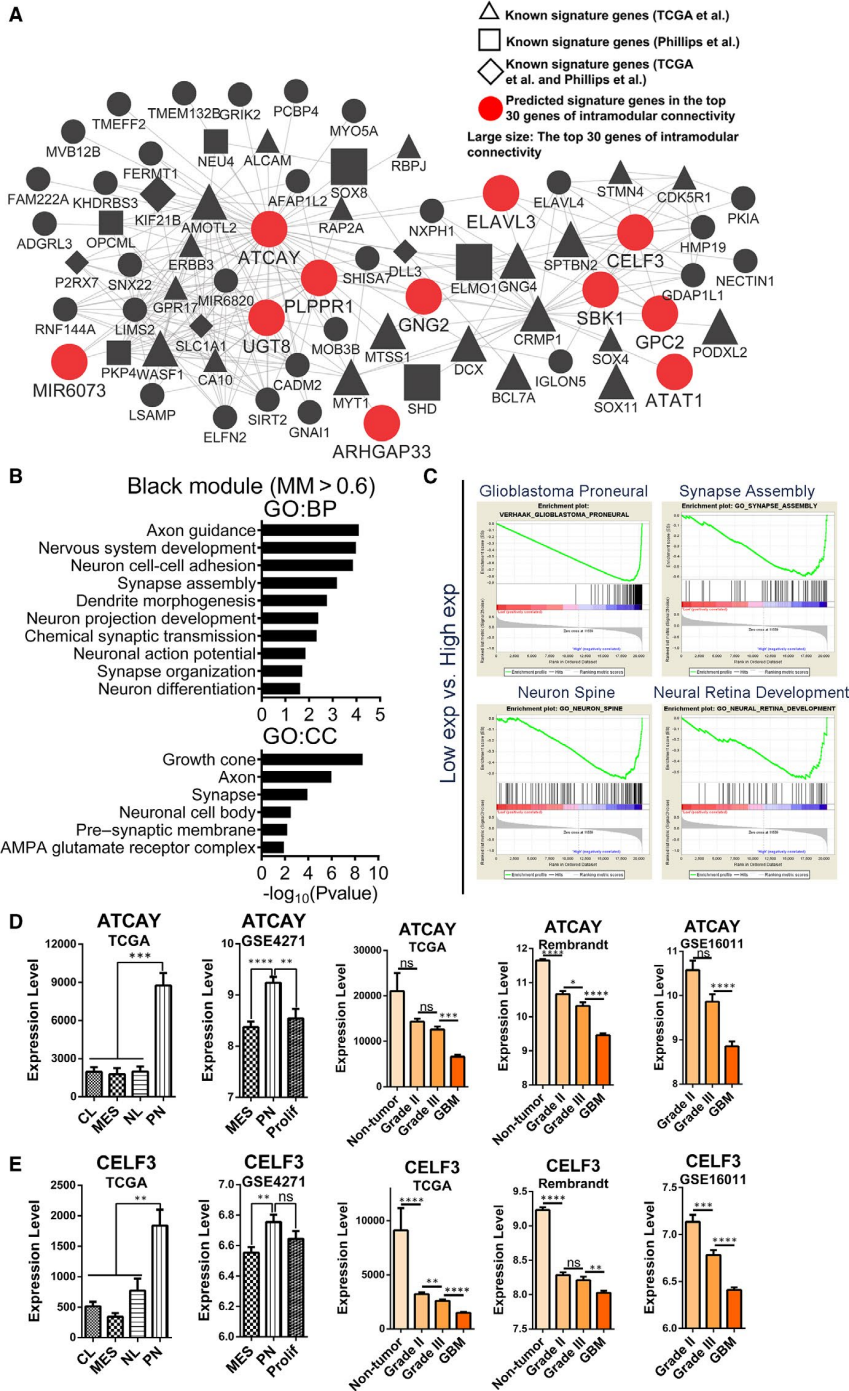
Functional annotation for black module and identification of candidate PN signature genes. (A) Network depiction of black module. Nodes correspond to genes, and lines correspond to connections, with the top 250 connections in black module shown. The red, large‐sized nodes are candidate PN signature genes. The nodes labelled with the letter ‘R’ or ‘C’ were radioresistant or chemoresistant genes. (B) Results of GO enrichment analysis for genes with MM > 0.6 in black module are shown. The GO terms are divided into two groups: BP and CC. (C) GSEA shows that several gene sets enriched in samples with high expression levels of ATCAY. (D) mRNA expression levels of ATCAY among different molecular subtypes and glioma grades in several data sets. (E) mRNA expression levels of CELF3 among different molecular subtypes and glioma grades in several data sets. Means ± SEM; one‐way ANOVA with Tukey's post hoc test; ns, *P* > .05, *, *P* < .05; **, *P* < .01, ***, *P* < .001, ****, *P* < .0001. GO, gene ontology; BP, biological process; CC, cellular component

The black module was identified as the PN subtype‐related module in previous results (Figure 1B). To better annotate the module function, we first selected 124 central genes whose MM > 0.6 (Table S2), including 55 known PN signature genes^4^, ^5^ and 69 new genes. GO enrichment analyses, using the 124 genes, showed enrichment for several GO terms that were functionally associated with neuronal development/maturity and structures of neurons (Figure 2B and Table S3), which were consistent with the previous studies.^4^, ^5^ Then, the top 250 connections in the black module were visualized as a network (Figure 2A). The known PN signature genes were labelled with different shapes. The hub genes labelled with large‐size nodes were defined as the top 30 genes of intramodular connectivity, which represent the most central genes in the co‐expression network in the module (Figure 2A and Table S2). The hub genes labelled as large, red, round nodes were identified as the predicted PN signature genes (Figure 2A and Table S2).

The genes with higher intramodular connectivity better represent the module. To identify the potential functions of these genes, we selected the top three genes of intramodular connectivity (ATCAY, CRMP1 and PLPPR1) to perform GSEA. The gene sets that were enriched in samples with high expression levels of the three genes are shown in Table S4. Furthermore, the intersections of these enriched gene sets were more representative of the functions of the hub genes, which are also shown in Table S4. The gene set of ‘Verhaak glioblastoma proneural’ and gene sets focused on the neuronal development and nervous system development were enriched in the samples containing any of the three highly expressed genes (Figure 2C and Table S4).

To validate these candidate PN signature genes, we compared the differential expression of these genes between various subtypes in two independent data sets (TCGA and http://www.ncbi.nlm.nih.gov/geo/query/acc.cgi?acc=GSE4271 data sets). Many of these gene expression levels were significantly higher in the PN subtype relative to the other subtypes (Figure 2D and 2E, Figure S4A). In addition, the expression levels of four of these genes (ATCAY, CELF3, ELAVL3 and UGT8) were highest in normal brain tissues, and negatively correlated with glioma grades (Figure 2D and 2E). The expression patterns of other hub genes are shown in Figures S4A and S4B.

### 3.4 Radioresistant/chemoresistant gene signatures only exist in Verhaak et al PN subtype

**Figure 3:**
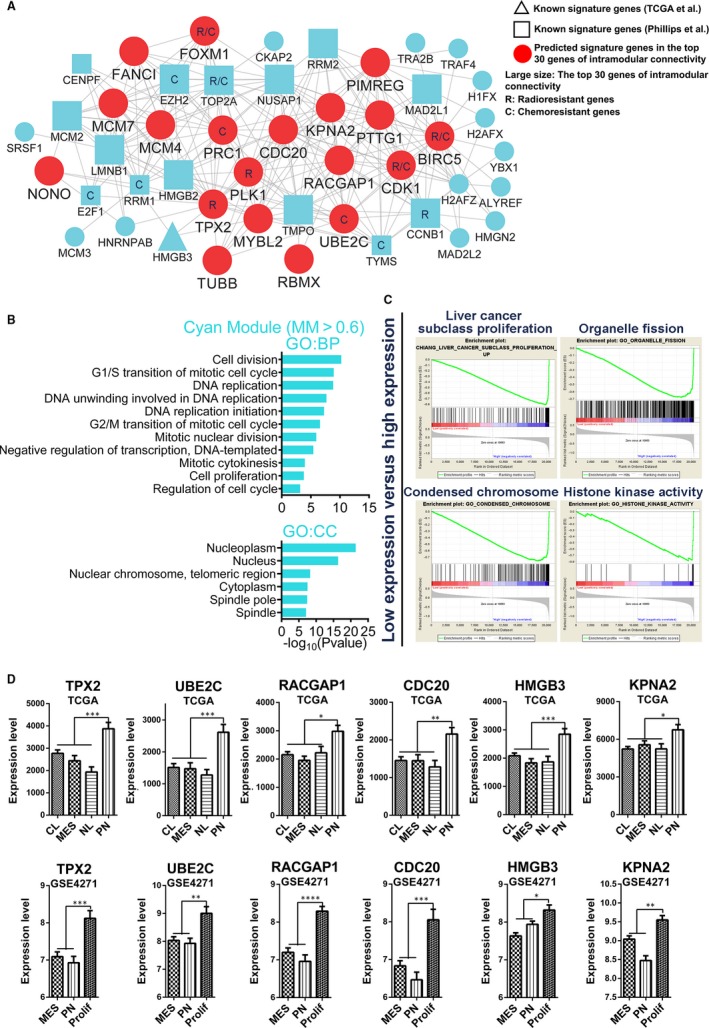
Functional annotation for cyan module and identification of candidate PN signature genes. (A) Network depiction of cyan module. Nodes correspond to genes, and lines correspond to connections, with the top 250 connections in cyan module shown. (B) GO enrichment analysis results of cyan module are shown. The GO terms are divided into two groups: BP and CC. (C) GSEA shows that some gene sets enriched in samples with high expression levels of TPX2. (D) mRNA expression levels of six genes (TPX2, UBE2C, RACGAP1, CDC20, HMGB3 and KPNA2) among molecular subtypes in two independent data sets (TCGA and http://www.ncbi.nlm.nih.gov/geo/query/acc.cgi?acc=GSE4271 data sets). Means ± SEM; one‐way ANOVA with Tukey's post hoc test; ns, *P* > .05, *, *P* < .05; **, *P* < .01, ***, *P* < .001, ****, *P* < .0001

The cyan module was found to be another PN subtype‐specific module (Figure 1B). As before, we selected 59 central genes with MM > 0.6 in the cyan module and found that there were 16 Prolif subtype signature genes from Phillips et al^4^ and 2 PN signature genes from Verhaak et al,^5^ which indicated that the cyan module could be a highly Prolif subtype–specific module. GO enrichment analysis showed that cycle/proliferation and structures of nucleus were enriched (Figure 3B and Table S3).

As before, the top 250 connections were visualized as a network (Figure 3A). To identify the potential functions of these genes, the top 3 hub genes (TPX2, CDC20 and UBE2C) were selected to perform GSEA (Tables S2 and S4). The results of GSEA, as shown in Figure 3C, suggest that the major functions of the cyan module were focused on cell proliferation and cell cycle (Figure 3C and Table S4).

To validate these predicted signature genes, we compared the differential expression of these genes between the different subtypes. We found that these genes were highly expressed in the Verhaak et al PN subtype relative to other subtypes in TCGA data set (Figure 3D and Figure S5A). However, in the Phillips et al data set (http://www.ncbi.nlm.nih.gov/geo/query/acc.cgi?acc=GSE4271), the expression levels of these genes were significantly higher in the Prolif subtype than the PN and MES subtypes (Figure 3D and Figure S5A).

With this, we identified two groups of genes with distinct functions (neuron development and cell cycle/proliferation) from black and cyan modules as the signature genes for Verhaak et al PN subtype. The group of genes involved in neuron development could be signature genes for the Phillips et al PN subtype (Figure 2D,2E and Figure S4A). However, the group of genes associated with cell cycle/proliferation were signature genes for the Phillips et al Prolif subtype (Figure 3D and Figure S5A). Thus, the Verhaak et al PN subtype and the Phillips et al PN subtype do not correspond exactly. However, there are overlaps between the Verhaak et al PN subtype and the Phillips et al Prolif subtype.

Interestingly, we found that there were numerous known radioresistant/chemoresistant genes that were occupied at important positions of the cyan module, such as TYMS, RRM1, FOXM1, TOP2A, CDK1, CCNB1 and BIRC5 (Figure 3A). Based on these results, the cyan module was also a radioresistance/chemoresistance‐related module, which may explain why the Verhaak et al PN subtype GBMs did not significantly benefit from radiotherapy and temozolomide.^5^ In addition, we speculate that the Prolif subtype, but not the Phillips et al PN subtype, could tolerate radiotherapy and temozolomide.

### 3.5 The tumour cells in MES subtype GBMs are under ER stress

**Figure 4:**
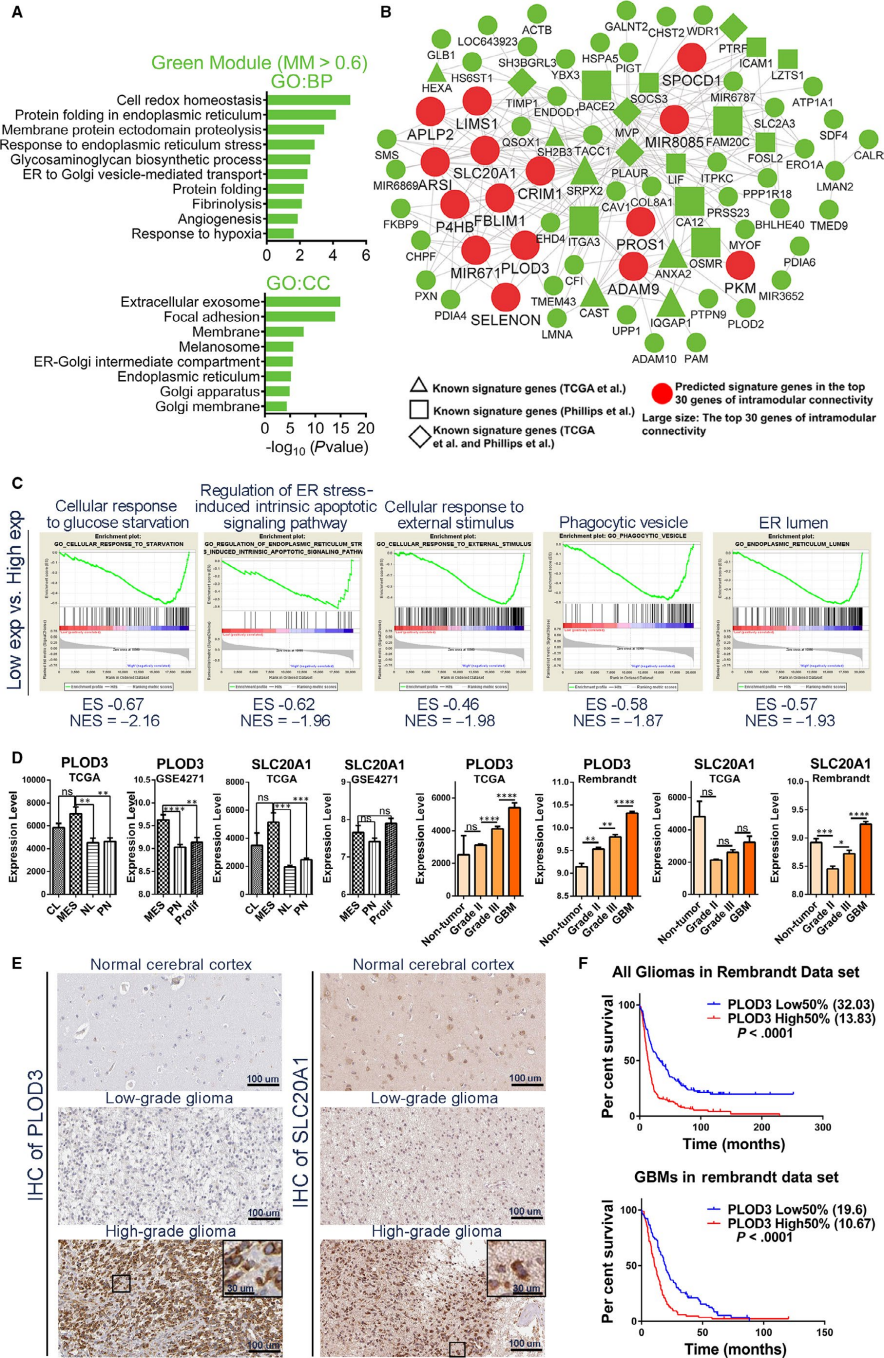
Functional annotation and network analyses for green module. (A) GO enrichment analysis results of green module are shown. The GO terms are divided into two groups: BP and CC. (B) Network depiction of green module. Nodes correspond to genes, and lines correspond to connections, with the top 250 connections in the green module shown. (C) GSEA results show that several gene sets enriched in GBMs with high expression levels of SRPX2. (D) mRNA expression levels of PLOD3 and SLC20A1 among molecular subtypes and glioma grades in different data sets. (E) IHC staining shows the protein expression levels and cellular localizations of PLOD3 and SLC20A1 in normal brain tissues, LGGs and HGGs. (F) Survival analyses between high 50% and low 50% PLOD3 expression group of all gliomas or GBMs in Rembrandt data set. Means ± SEM; one‐way ANOVA with Tukey's post hoc test; ns, *P* > .05, *, *P* < .05; **, *P* < .01, ***, *P* < .001, ****, *P* < .0001. IHC, immunohistochemistry; LGGs, low‐grade gliomas; HGGs, high‐grade gliomas

The green module was identified as the MES subtype–specific module in previous results (Figure 1B). GO analysis indicated that the major functions of the green module could be focused on the ER stress response (ERSR; Figure 4A and Table S3).

As before, the 15 large, red round nodes in the network were identified as candidate MES signature genes (Figure 4B and Table S2). GSEA of the top 3 hub genes (SRPX2, ITGA3 and FAM20C) was performed (Figure 4C and Table S4). These results showed that the tumour cells in the MES subtype GBM were exposed to stress conditions, such as ischaemia or hypoxia, which lead to ER stress (ERS), resulting in the unfolded protein response (UPR), regulation of cell apoptosis and autophagy levels.^15^

In the green module, we found that there were numerous genes reported to be involved in the response to stress conditions, such as HSPA5, ADAM9, ADAM10, PDIA3, PDIA6 and CAV1. We also found that several genes associated with autophagy were in the green module. Autophagy plays an important role in the ER stress response, which was reduced in the MES subtype (discussed in Discussion section).

In addition, we found that a majority of the 15 predicted genes were highly expressed in the MES subtype, relative to other subtypes. We also found that most of these genes were highly expressed in GBMs, relative to grade III gliomas (Figure 4D, Figures S6A, and S6B and C). Furthermore, the expression levels of several genes (PLOD3, SLC20A1, ADAM9, FBLIM1, SPOCD1, P4HB, PROS1 and SELENON) were positively correlated with glioma grades (Figure 4D and Figure S6B). Immunohistochemical (IHC) stainings showed that the expression trends of PLOD3 and SLC20A1 were consistent with the trends of RNA levels (Figure 4E). For PLOD3, there are 3 LGGs (three negative intensity) and 9 HGGs (one strong, two moderate, 4 weak and two negative intensity). For SLC20A1, there are 3 LGG (two moderate and 1 weak intensity) and 8 HGG (one strong, six moderate and one negative intensity) (Table S1).

We further wonder whether PLOD3 or SLC20A1 expression could affect overall patient survival. In the Rembrandt, TCGA or http://www.ncbi.nlm.nih.gov/geo/query/acc.cgi?acc=GSE16011 data sets, all glioma or GBM patients were divided into high and low 50% expression groups, according to their expression levels. High 50% expression group had poorer survival than low 50% expression group (Figure 4F and S6D).

### 3.6 Uncovering molecular mechanisms for higher necrosis proportion of MES subtype

**Figure 5:**
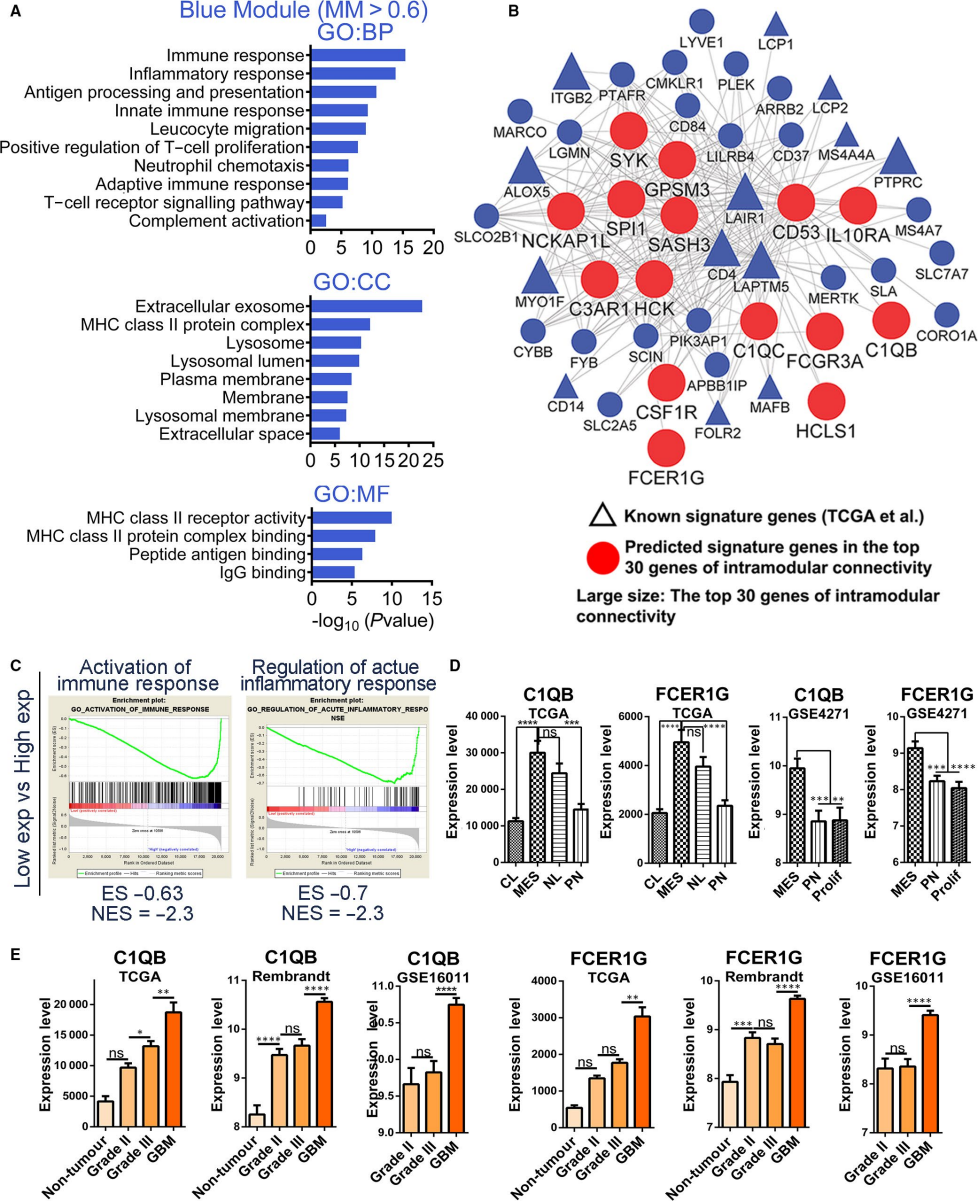
Functional annotation and network analyses for blue module. (A) GO enrichment analysis results of blue module are shown. The GO terms are divided into three groups: BP, CC and MF. (B) Network depiction of blue module. Nodes correspond to genes, and lines correspond to connections, with the top 250 connections in blue module shown. (C) GSEA results show that several gene sets enriched in GBMs with high expression levels of CD53. (D) mRNA expression levels of C1QB and FCER1G among molecular subtypes in TCGA and http://www.ncbi.nlm.nih.gov/geo/query/acc.cgi?acc=GSE4271 data sets. (E) mRNA expression levels of C1QB and FCER1G among different glioma grades in TCGA, Rembrandt and http://www.ncbi.nlm.nih.gov/geo/query/acc.cgi?acc=GSE16011 data sets. Means ± SEM; one‐way ANOVA with Tukey's post hoc test; ns, *P* > .05, *, *P* < .05; **, *P* < .01, ***, *P* < .001, ****, *P* < .0001

The blue module was also an MES subtype–specific module (Figure 1B). As before, we selected 204 central genes with MM > 0.6 in the blue module and revealed that there were 44 MES subtype signature genes from Verhaak et al^5^ and 6 MES subtype signature genes from Phillips et al^4^ The GO analysis revealed that the immune inflammatory response–related term was significantly enriched (Figure 5A and Table S3), which is consistent with the increased proportion of necrosis found in the MES subtype.^5^

We found that there were many immune inflammatory response–related genes in the module (Figure 5B and Table S2), such as CD53/CD4/CD14/CD37/CD300a/CD74/CD163/CD84, C1QA/C1QB/C1QC, IL10RA/IL13RA1, FCER1G/FCGR3A, HLA‐DMA/HLA‐DMB/HLA‐DMB1/HLA‐DOA/HLA‐DRA/HLA‐DPB1/HLA‐DQA1/HLA‐DQB1/HLA‐DPA1, LAIR1, SASH3, HCK, SPI1, C3AR1, which could be molecular mechanisms underlying the higher proportion of necrosis found in the MES subtype. The GSEA of top 3 hub genes (CD53,LAIR1 and LAPTM5) also showed that immune inflammatory response–related processes were enriched (Figure 5C and Table S4). We found that these genes were highly expressed in the MES subtype, relative to other subtypes (Figure 5D and Figure S5C). In addition, we revealed that most of these genes were up‐regulated in GBMs, relative to grade III gliomas (Figure 5E and Figure S5B). Moreover, we found that both C1QB and FCER1G had the highest expression levels in GBMs, as well as the lowest expression levels in normal brain tissue, and that the expression levels of C1QB and FCER1G were positively correlated with glioma grades (Figure 5E).

### 3.7 Uncovering molecular mechanisms for angiogenesis in MES subtype GBMs

**Figure 6:**
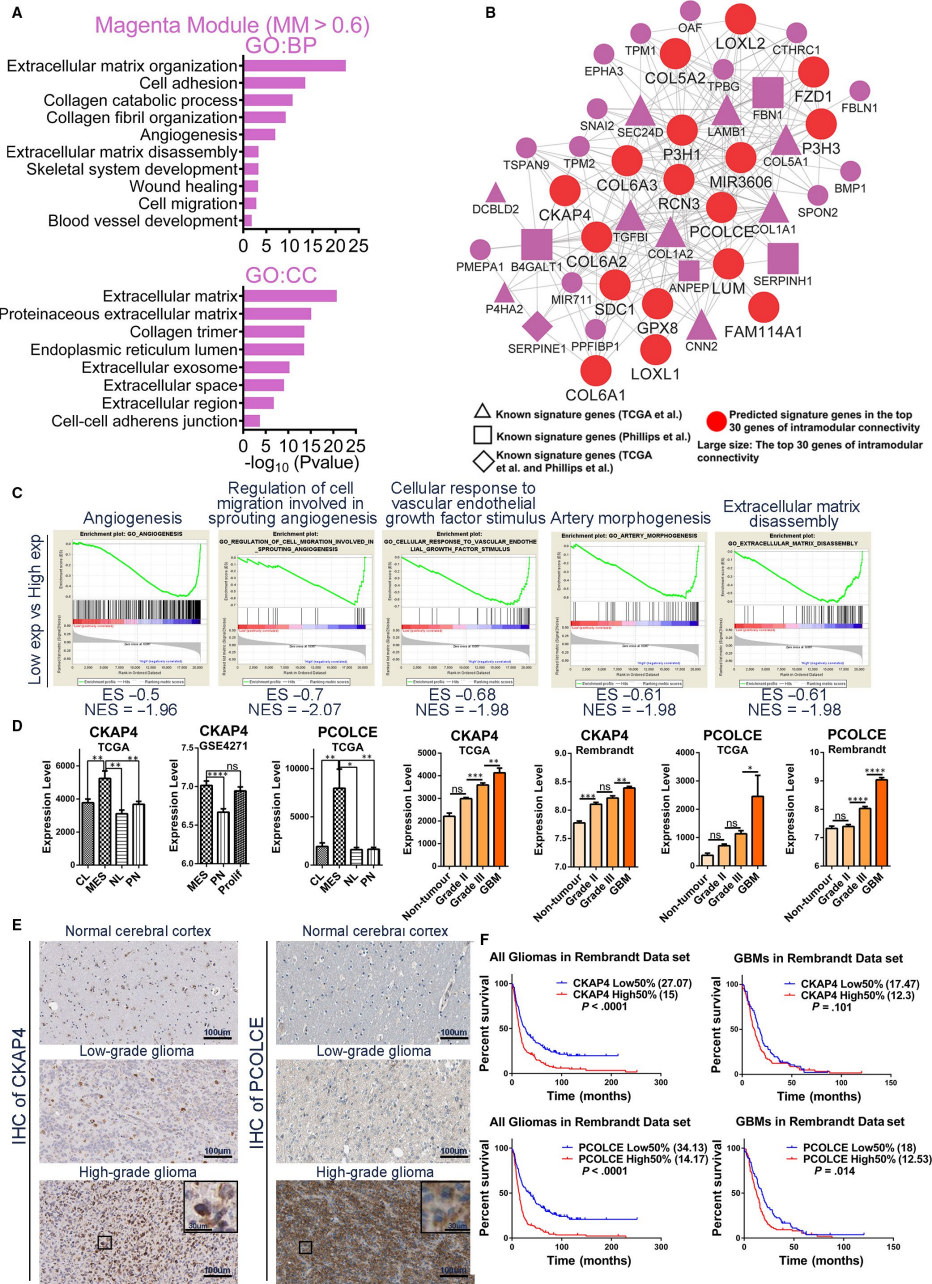
Functional annotation and network analyses for magenta module. (A) GO enrichment analysis results of magenta module are shown. The GO terms are divided into two groups: BP and CC. (B) Network depiction of magenta module. Nodes correspond to genes, and lines correspond to connections, with the top 250 connections in magenta module shown. (C) GSEA shows that several gene sets enriched in GBMs with high expression levels of RCN3. (D) mRNA expression levels of CKAP4 and PCOLCE among molecular subtypes and glioma grades in different data sets. (E) IHC staining shows the protein expression levels and cellular localizations of CKAP4 and PCOLCE in normal brain tissues, LGGs and HGGs. (F) According to the expression levels of CKAP4 and PCOLCE, survival analyses between high 50% expression group and low 50% expression group of all gliomas or GBMs in Rembrandt data set. Means ± SEM; one‐way ANOVA with Tukey's post hoc test; ns, *P* > .05, *, *P* < .05; **, *P* < .01, ***, *P* < .001, ****, *P* < .0001

The magenta module was also an MES subtype–specific module (Figure 1B). As before, we selected 96 central genes with MM > 0.6 in the magenta module, and revealed that there were 20 MES subtype signature genes from Verhaak et al^5^ and 9 MES subtype signature genes from Phillips et al^4^ The GO results suggested that significant angiogenesis and regulation of the collagen and extracellular matrix are shown in the MES subtype GBMs (Figure 6A and Table S3).

The 17 large, red round nodes in the network were identified as candidate MES signature genes (Figure 6B and Table S2). The GSEA results of the top 3 hub genes (RCN32, P3H1 and LAMB1) confirmed the important role of angiogenesis in the MES subtype (Figure 6C and Table S4). Moreover, extracellular matrix reorganization (disassembly and reorganization), collagen matrix reorganization, cell adhesion and cell migration are important for extracellular remodelling during angiogenesis or blood vessel development.^16^, ^17^

We found that there were several genes associated with angiogenesis (Figure 6B and Table S2), including COL18A1, NRP1, CYP1B1, ITGA5, TGFBI, SERPINE1, SHC1, ANPEP, ELK3, MMP14, MYH9, COL1A1, COL1A2, SDC1, RCN3 and CKAP4. Taken together, we identified the molecular mechanisms of the characteristic angiogenesis found in the MES subtype. Moreover, we found that the expression levels of predicted genes were significantly higher in the MES subtype and that the expression levels of these genes in the GBMs were higher than grade III gliomas in the three independent data sets (Figure 6D and Figures S7A and S6B). In addition, we found that several genes had the highest expression levels in GBMs and that the expression levels of these genes were positively correlated with the glioma grades (Figure 6D and Figure S7A). IHC staining showed that protein expression trends of CKAP4 and PCOLCE were consistent with the trends of RNA levels (Figure 6E). For CKAP4, there are 3 LGGs (three strong intensity) and 9 HGGs (six strong, two moderate and 1 weak intensity). Moreover, only 1 LGG showed more than 75% quantity of IHC staining, while 4 HGGs showed more than 75% quantity. For PCOLCE, there are 4 LGG (2 weak and two negative intensity) and 7 HGG (2 moderate and five negative intensity) (Table S1). We further wonder whether CKAP4 and PCOLCE expression could affect overall patient survival. In the Rembrandt data sets, all glioma or GBM patients were divided into high and low 50% expression groups according to their expression levels. High 50% expression group had significantly poorer survival than low 50% expression group (Figure 6F).

### 3.8 Genes involved in alternative RNA splicing are significantly up‐regulated in CL GBMs

**Figure 7:**
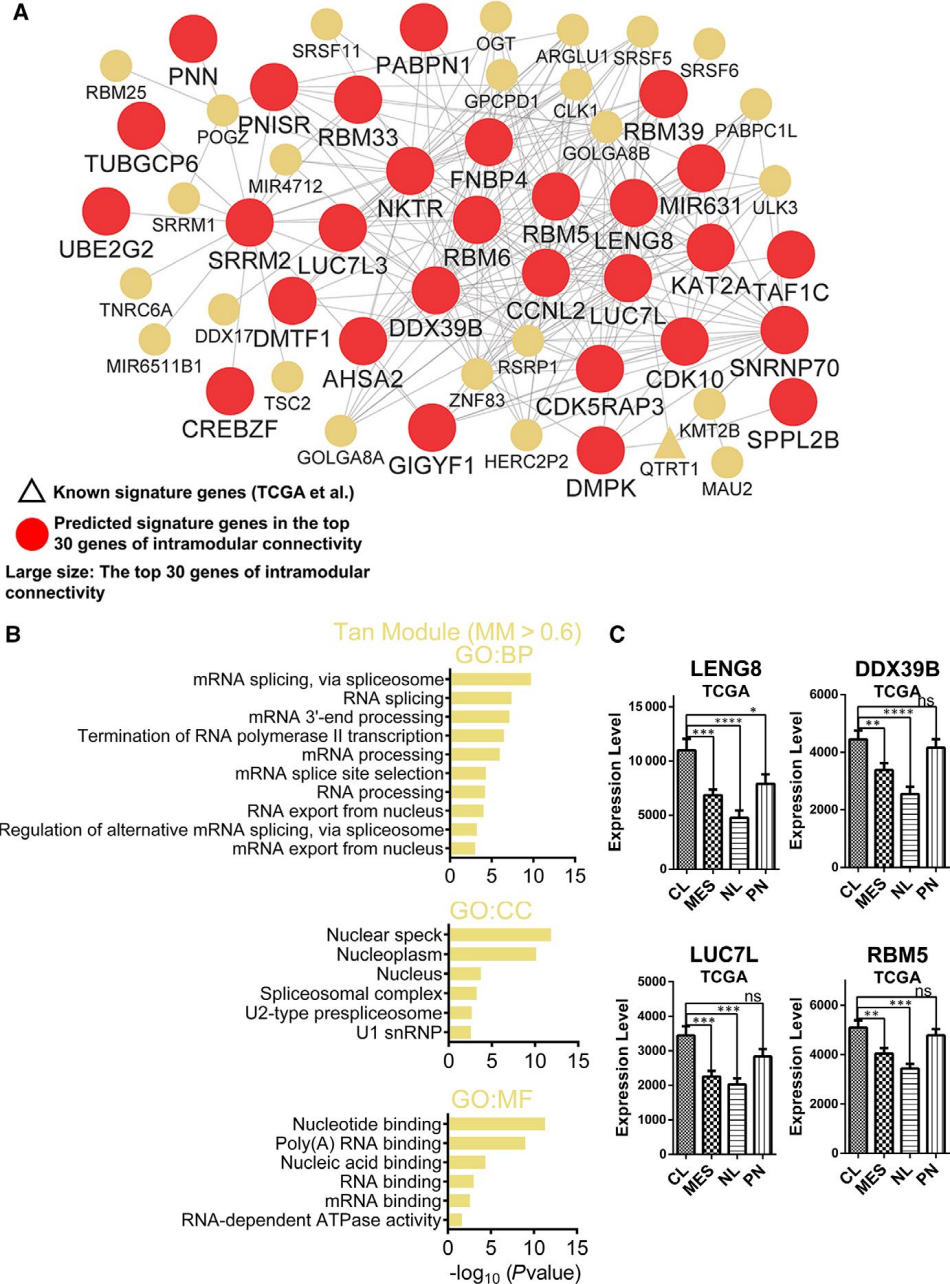
Functional annotation and network analyses for tan module. (A) Network depiction of tan module. Nodes correspond to genes, and lines correspond to connections, with the top 250 connections in the tan module shown. (B) GO enrichment analysis results of tan module are shown. The GO terms are divided into three groups: BP, CC and MF. (C) mRNA expression levels of four central genes (LENG8, DDX39B, LUC7L and RBM5) among molecular subtypes in TCGA data set. Means ± SEM; one‐way ANOVA with Tukey's post hoc test; ns, *P* > .05, *, *P* < .05; **, *P* < .01, ***, *P* < .001, ****, *P* < .0001

The tan module was identified as the CL subtype‐specific module in previous results (Figure 1B). GO analyses revealed that alterative RNA splicing‐related terms were significantly enriched. Nearly 53.3% of the genes (49 of 92 central genes in tan module) were enriched in the term of ‘nucleoplasm’ and ‘nucleus’ (Figure 7B and Table S3).

The 29 large, red, round nodes in the network were identified as candidate CL signature genes (Figure 7A and Table S2). We found that the RNA binding motif protein (RBM) family members (RBM5/6/25/33/39), SRRM1/2 and SRSF5/6/11, were in the central genes and played a role in the splicing and processing of mRNA. Moreover, two of the top 3 genes of intramodular connectivity (DDX39B and LUC7L) also serve important roles in alternative RNA splicing (Figure 7A and Table S2).^18^, ^19^

In addition, the expression patterns of these candidate CL signature genes were confirmed (Figure 7C and Figure S9A). Taken together, we first reported that the genes involved in RNA alternative splicing are significantly up‐regulated in the CL subtype, and we have identified several candidate CL signature genes.

### 3.9 Increasing energy synthesis in NL subtype GBMs

The turquoise module was identified as the NL subtype–specific module in previous results (Figure 1B). GO analyses revealed that energy metabolism–related terms were significantly enriched. Moreover, the enriched GO terms in the CC category primarily contained ‘mitochondrial inner membrane’, ‘mitochondrion’, ‘mitochondrial respiratory chain complex I’ and ‘mitochondrial matrix’. Nearly 22.5% of the genes (135 of 600 central genes in turquoise module) were enriched in the term of ‘mitochondrion’ (Figure S8B and Table S3).

The 18 large, red, round nodes in the network were identified as candidate NL signature genes. There were several protein families associated with mitochondrial energy synthesis in the central genes of the turquoise module, such as the ATP synthase family (ATP5A1/ATP5A1/ATP5E/ATP5F1/ATP5G1/ATP5G2/ATP5G3/ATP5H/ATP5I/ATP5J/ATP5J2/ATP5L/ATP5O/ATP6AP2/ATP6V0B/ATP6V0E1/ATP6V1E1/ATP6V1F/ATP6V1G1/ATPIF1), the mitochondrial ribosomal protein family (MRPL13/MRPL15/MRPL20/MRPL24/MRPL27/MRPL3/MRPL32/MRPL33/MRPL34/MRPL43/MRPL49/MRPL51/MRPL52/MRPL54/MRPL57/MRPS15/MRPS18B/MRPS21/MRPS23/MRPS33/MRPS35/MRPS7) and the NADH:ubiquinone oxidoreductase family, (NDUFA1/NDUFA2/NDUFA4/NDUFA5/NDUFA6/NDUFA9/NDUFA11/NDUFA12/NDUFA13/NDUFAB1/NDUFB1/NDUFB2/NDUFB3/NDUFB4/NDUFB5/NDUFB6/NDUFB7/NDUFB9/NDUFB10/NDUFC2/NDUFS3/NDUFS4/NDUFS5) (Figure S8A and Table S2). Taken together, we found that the levels of mitochondrial activity and energy synthesis are significantly higher in the NL subtype.

In addition, the expression levels of the 18 candidate NL signature genes were compared between different subtypes (Figures S8C and S9B), which further confirmed that these genes could be candidate NL signature genes.

## 4 DISCUSSION

The classic histopathological grading is the main stratification method applied in prognostic prediction and risk in management decisions. However, the classic histopathology is subjective and cannot objectively, systematically and accurately reflect the genetic background and biological characteristics of glioma tissue. At the same time, using classic histopathology has low efficacy in guiding treatment and predicting clinical prognosis of patients with glioma. Some LGGs have the potential to show poor clinical outcome such as HGGs, whereas some HGG patients could achieve long‐term survival.

Phillips et al identified three molecular subtypes of high‐grade astrocytomas, according to the gene expression profiling: PN, MES and Prolif.^4^ In 2010, Verhaak et al classified GBMs into four molecular subtypes: CL, NL, MES and PN, based on transcriptome data.^5^ Yan et al^20^ subclassified the gliomas into three subgroups, according to their gene signature: G1, G2 and G3, which were enriched with PN, NL and the MES subtypes, respectively. Although the molecular subtypes have varied in different studies, the MES and PN subtypes appear robust and consistent among the various classifications.^4^, ^5^, ^20^ Glioma patients in the MES subtype exhibit a poorer prognosis compared to the PN subtype, which may be related to the fact that the PN subtype gliomas are more frequently associated with increased IDH mutations, glioma‐CpG island methylator phenotypes (G‐CIMP) and 1p/19q co‐deletions.^5^ However, the PN subtype is characterized by unresponsiveness to chemical and radiation therapy.^3^, ^5^ In addition, intratumour heterogeneity describes the diversity in individual tumours, which has become a research hot spot in recent years. By using the single GBM cell RNA sequencing, a study by Patel et al^21^ suggested that GBMs are comprised of a mixture of tumour cells with variable GBM subtype footprints. Wang et al^22^ found that the bulk of the GBM samples were classified into the same primary subtype as the majority of their single tumour cells. GBMs are highly heterogeneous, so it is important to uncover the pathological processes and molecular mechanisms of the various molecular subtypes of GBMs.

In the cyan module, we found that the core genes with the highest intramodular connectivity are associated with either radioresistance or chemoresistance, such as TPX2 microtubule nucleation factor (TPX2), ubiquitin‐conjugating enzyme 2C (UBE2C) and polo‐like kinase 1 (PLK1) (Figure 3A and Table S2). TPX2 plays an important role in spindle assembly^23^ and is overexpressed in several tumours, including breast cancer, hepatocellular carcinoma and squamous cell lung cancer.^24^ Recent research revealed that the TPX2 expression levels were elevated in radioresistant squamous carcinoma cells of the lung, relative to the corresponding parental cells, and that the overexpression of TPX2 induced the radioresistance found in the NCI‐H226 cell line in vitro and in vivo.^24^ In addition, a previous study revealed that inhibition of UBE2C in breast cancer cells could decrease cell proliferation and increase radiosensitivity and chemosensitivity for doxorubicin, tamoxifen and letrozole.^25^ Furthermore, PLK1 inhibition also sensitized medulloblastoma cells to radiation and decreased cell proliferation.^26^

We found that tumour cells in the MES subtype GBMs may be exposed to hypoxia, ischaemia, hypoglycaemia, etc, and that the ER stress response is induced within the tumour cells of the MES subtype GBMs. Hypoxia‐inducible factor‐1α (HIF1A), the required subunit of HIF1, which is the main transcriptional factor in hypoxia and plays an essential role in tumour angiogenesis and proliferation,^27^ was also in the green module, and significantly up‐regulated in the MES subtype (Table S2). Moreover, we found that HSPA5, which has a crucial role in the ER stress response, was one of the central genes in the green module. HSPA5 can bind and deactivate three primary ER transmembrane receptors (IRE1α, ATF6 and PERK) in the absence of stress.^28^ The three ER transmembrane receptors can function as molecular sensors to detect the accumulation of unfolded proteins.^29^ As the unfolded proteins accumulate, HSPA5 releases the ER transmembrane receptors for activation of UPR, and binds to the hydrophobic domains of unfolded proteins to reduce cell damage.^30^ Thus, harmful environmental stress, such as ischaemia or hypoxia, induces low intracellular glucose levels, thereby activating HSPA5 and further inducing UPR, which leads to pro‐survival outcome of cells under stress.^31^

ADAM9 was the most central gene in the green module (Figure 4B and Table S2), which can be induced under stress and further promote angiogenesis in tumours. ADAM9 can cleave and release several growth factors (heparin‐binding EGF‐like growth factor (HB‐EGF), epidermal growth factor (EGF) and fibroblast growth factor receptor 2IIIB (FGFR2IIIB)), as well as degrade the extracellular matrix substrates and interact with crucial regulatory factors (mitotic arrest deficient 2 β (MAD2β), SH3PX1 and SH3GL2^32^) through its different domains. Moreover, previous studies have found that overexpression of ADAM9, induced by ROS, could increase the shedding of several membrane proteins (EphB4, Tie‐2, CD40, VCAM, Flk‐1 and VE‐cadherin) from endothelial cells to enhance pathological neovascularization^33^ and that ADAM9 could up‐regulate VEGFA, ANGPT2 and PLAT to promote vascular remodelling in lung cancer cells.^34^ We believe that ADAM9, the most representative gene for green module, could significantly participate in promoting angiogenesis in the MES GBMs and could serve as a potential therapeutic target.

A previous study revealed that the autophagic activity in the MES glioma stem‐like cells (GSCs) was higher than that in PN GSCs, which was also attributed to increased tumorigenicity and therapy resistance of MES GSCs.^35^ Consistent with this finding, we also found that the autophagic activity was increased in the MES GBMs, when compared with the other subtypes. In this study, we found that CAV1 was one of the central genes in the green module (Figure 4B and Table S2) and was significantly up‐regulated in the MES GBMs. Overexpression of CAV1 in hypoxic cells can increase tyrosine phosphorylation of EGFR and reduce the hypoxia‐induced autophagy that occurs after 48h of hypoxia, thereby altering the response from cell death to cell survival.^36^ We believe that CAV1 may regulate autophagic activity to cope with the hypoxic microenvironment in MES GBMs, and could serve as a potential therapeutic target.

In the magenta module, we found that there were several crucial angiogenesis‐related genes. SERPINE1, one of the central genes in the magenta module, can suppress integrin α_v_β_3_ (ITGAV and ITGB3)–mediated cell adhesion to vitronectin, but will facilitate integrin α_5_β_1_ (ITGA5 and ITGB1)–mediated migration from vitronectin to fibronectin (Figure S6B and Table S2).^37^ Moreover, SERPINE1 can promote the migration of endothelial cells from their perivascular space, which contains vitronectin to the fibronectin‐rich tumour tissues, and further facilitate angiogenesis within the tumour, thereby contributing to the angiogenesis within the MES subtype.^37^ Interestingly, both ITGA5 and ITGB1 belong to central genes in the magenta module, which were also significantly up‐regulated in the MES subtype (Table S2). Extracellular matrix remodelling is essential for the activation and migration of endothelial cells that contribute to angiogenesis. In addition, we also found several extracellular matrix components in the central genes of the magenta module, including the collagen superfamily, such as COL1A1, COL1A2, COL18A1, COL6A1, COL6A2, COL6A3, COL5A1, COL5A2, COLGALT1 (Figure S6B and Table S2). These genes could play important roles in the progression of tumour angiogenesis.^38^, ^39^ MMP14, one of the central genes in the magenta module (Table S2), is a transmembrane metalloproteinase that plays an important role in angiogenesis through several steps, including extracellular matrix degradation, endothelial cell invasion, migration into surrounding tissue, formation of capillary tubes, deposition of a new basement membrane and recruitment of accessory cells.^40^ Moreover, in human GBM and breast cancer xenograft models, MMP14 overexpression can up‐regulate VEGF expression and therefore promote angiogenesis.^41^ In this study, we revealed several key genes (SERPINE1, ITGA5, ITGB1, MMP14 and collagen superfamily) that regulate angiogenesis in MES GBMs, which could be potential therapeutic targets.

Previous studies have shown that a more serious immune/inflammatory response is present within the MES subtype GBMs. Prins et al^42^ revealed that the MES subtype GBMs have a better response to immunotherapy, implying that this subtype may be more immunogenic. In addition, several studies revealed that there are increased numbers of tumour‐infiltrating lymphocytes or microglia/macrophage present in the MES subtype GBMs, when compared with the other subtypes of GBM.^43^, ^44^ NF1 was frequently deactivated through genomic copy number loss or somatic mutations in MES subtype GBMs.^5^ Wang et al found that NF1 deactivation may promote microglia/macrophage recruitment in GBMs.^22^ In this study, we used a distinct and powerful system biology analysis method (WGCNA) to identify the immune/inflammatory response related to each module based on the co‐expression relationships between all expressed genes across samples, and found that modules are highly correlated with the MES subtype GBMs. On the one hand, we confirmed the conclusions of other studies. On the other hand, we have revealed several key regulatory genes in the immune/inflammatory response of MES subtype GBMs.

In addition, we found that the genes associated with alternative RNA splicing are significantly up‐regulated within the CL subtype GBMs. Alternative splicing of pre‐mRNA is a crucial regulator of gene expression, as it can generate numerous transcripts from a single protein‐coding gene. Recently, numerous studies have suggested that dysregulation of alternative RNA splicing plays an important role in tumour progression.^45^ We speculated that alternative RNA splicing could also play an essential role in the CL subtype GBMs, which warrants further research. Changes in energy metabolism can be a cause of tumorigenesis.^46^

In previous studies, it was found that the NL subtype was associated with tumour margins, where increased normal neural tissue could be detected.^47^, ^48^ Another study suggested that the NL subtype is non–tumour‐specific.^22^ However, in this study, we still attempted to analyse the acquired NL‐specific module and found that the increased energy production may play an important role in the NL subtype, which also requires further research.

## CONFLICT OF INTEREST

The authors declare no conflicts of interest.

## AUTHOR CONTRIBUTIONS

JZ, AS and JW initiated the project. AS, JW and Y.‐BP conceived and designed the research plans. Y.‐BP, SW, BY and ZJ performed the WGCNA. SW and Y.‐BP helped with the IHC data analysis. Y.‐BP helped with the survival analysis. ZJ helped with GO analysis. Y.‐BP, SW and BY co‐wrote the paper. CL assisted with language polishing. All authors discussed the results and commented on the manuscript.

## ETHICAL APPROVAL

All procedures performed in studies involving human participants were in accordance with the ethical standards of the institutional and/or national research committee and with the 1964 Helsinki declaration and its later amendments or comparable ethical standards.

## INFORMED CONSENT

As personal identifying information was not included in the Rembrandt, TCGA and HPA database, the informed consent was not required in this study.

## Data Availability

All data needed to evaluate the conclusions in the paper are present in the paper and/or the Supplementary Materials. Additional data related to this paper may be requested from the authors.
