# Supplementary figures and images for: Transcriptome analyses reveal molecular mechanisms underlying phenotypic differences among transcriptional subtypes of glioblastoma
Source: J Cell Mol Med. 2020 Feb 24;24(7):3901–16. doi: 10.1111/jcmm.14976 (PMC7171397; doi:10.1111/jcmm.14976)

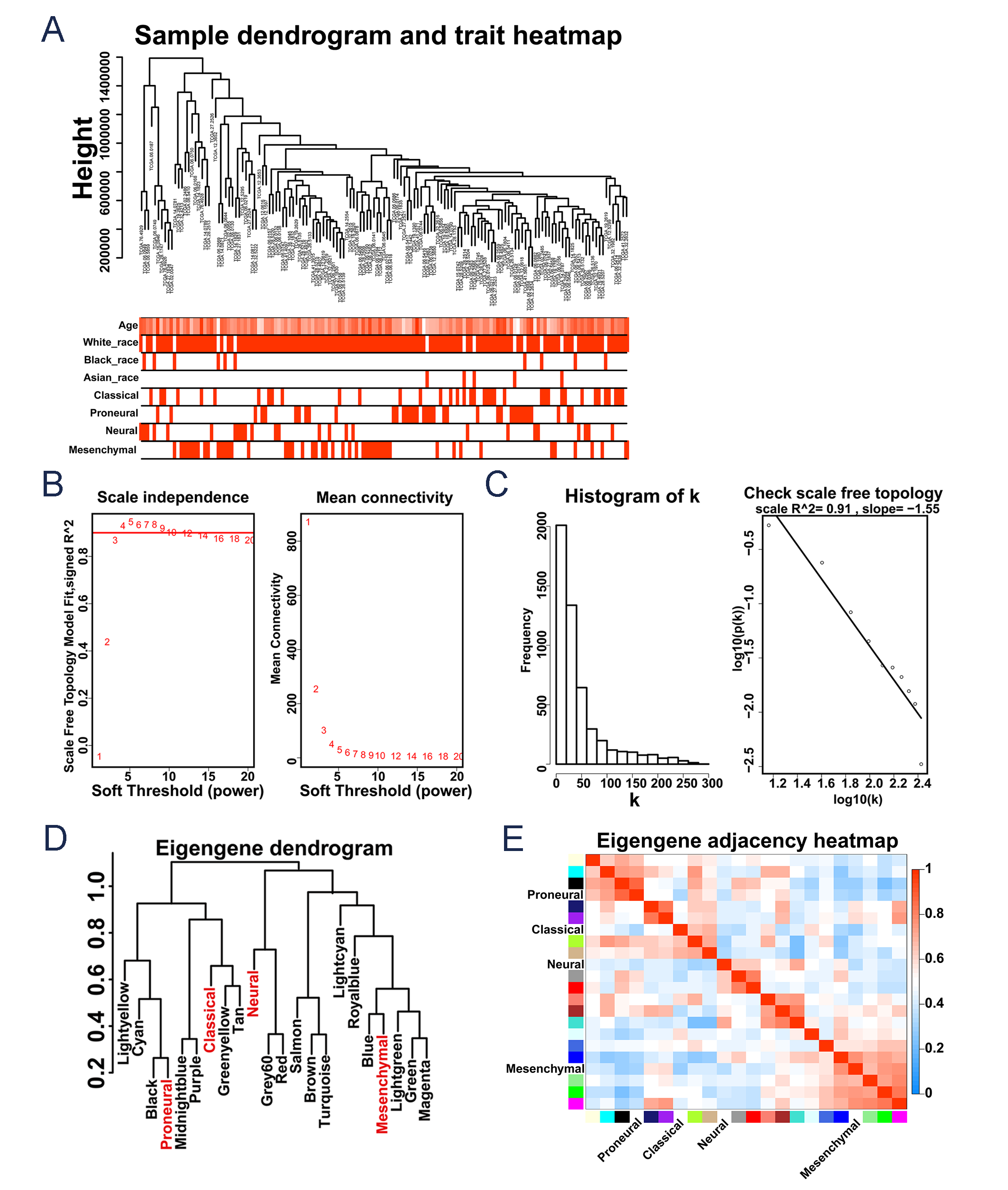

Supplement: Supplementary file 1 [file JCMM-24-3901-s001.tif]

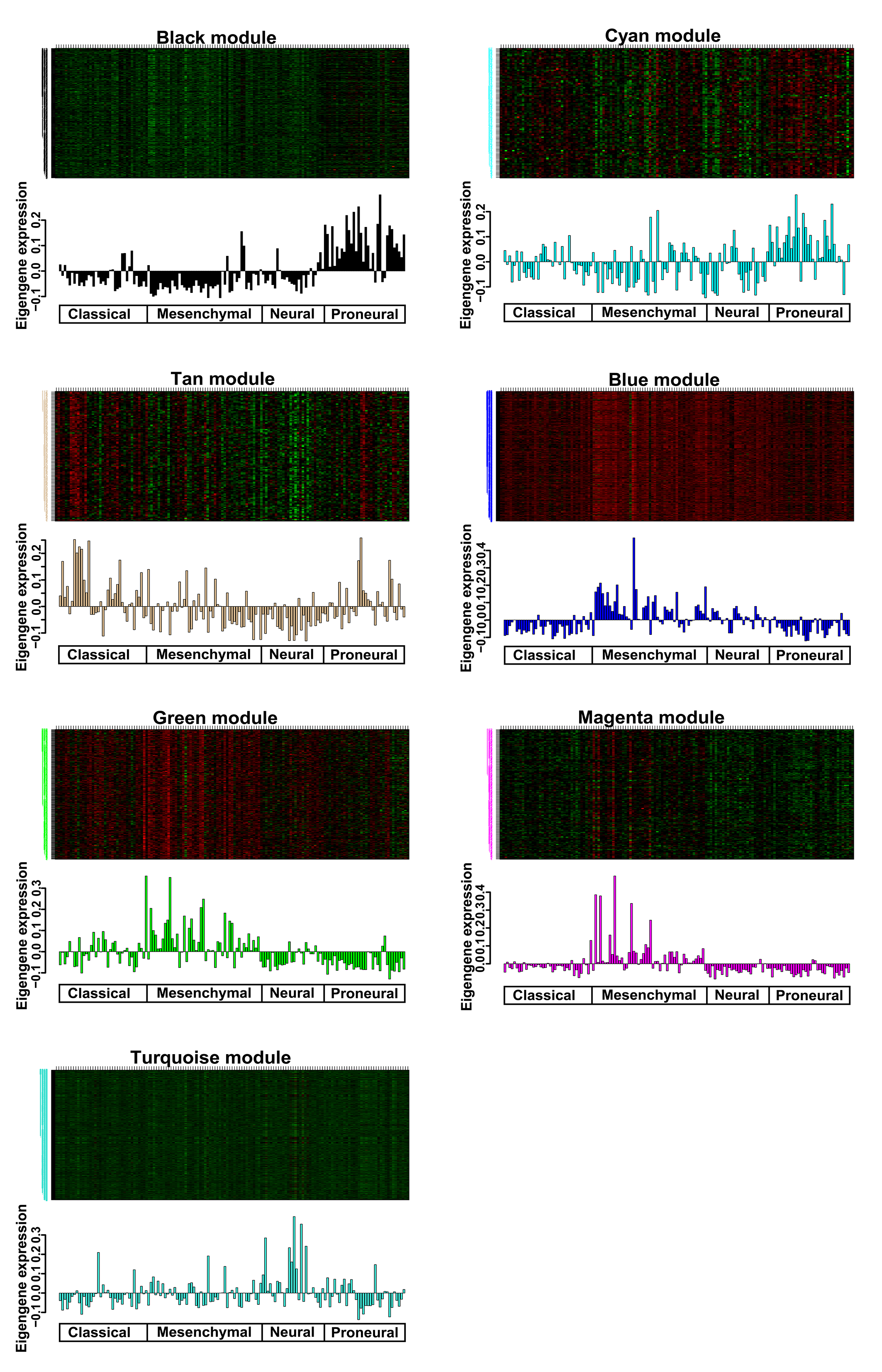

Supplement: Supplementary file 2 [file JCMM-24-3901-s002.tif]

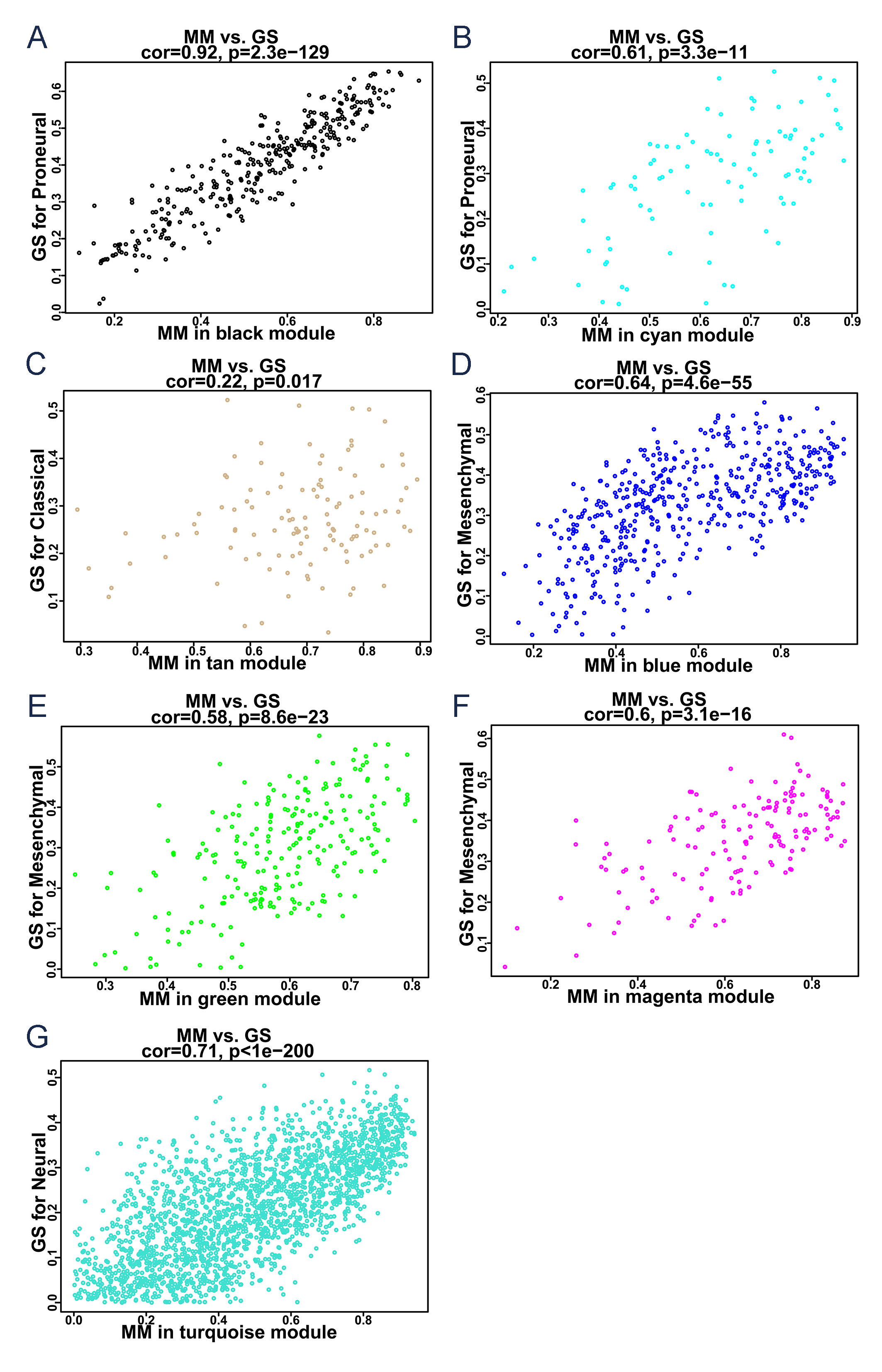

Supplement: Supplementary file 3 [file JCMM-24-3901-s003.tif]

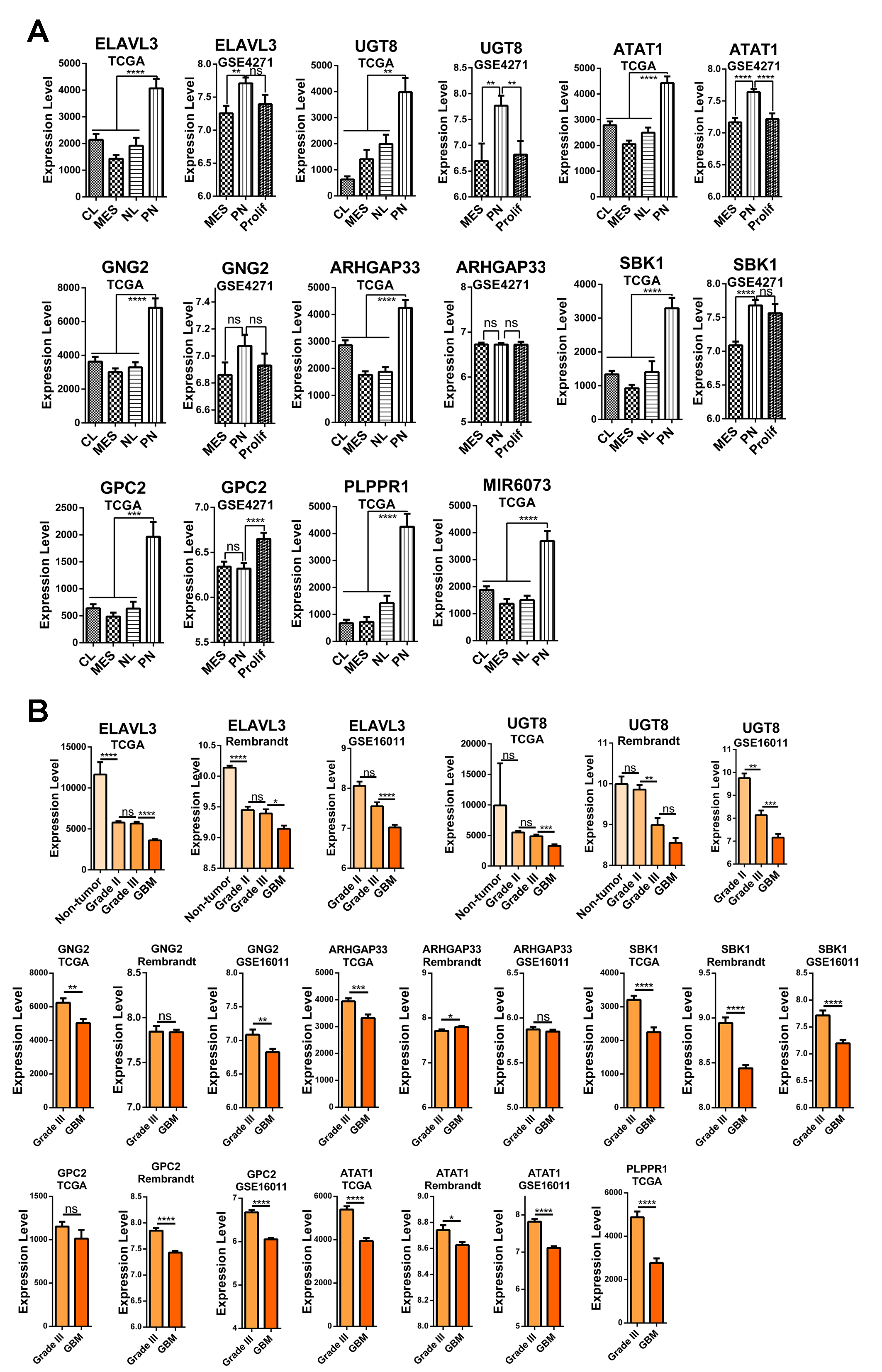

Supplement: Supplementary file 4 [file JCMM-24-3901-s004.tif]

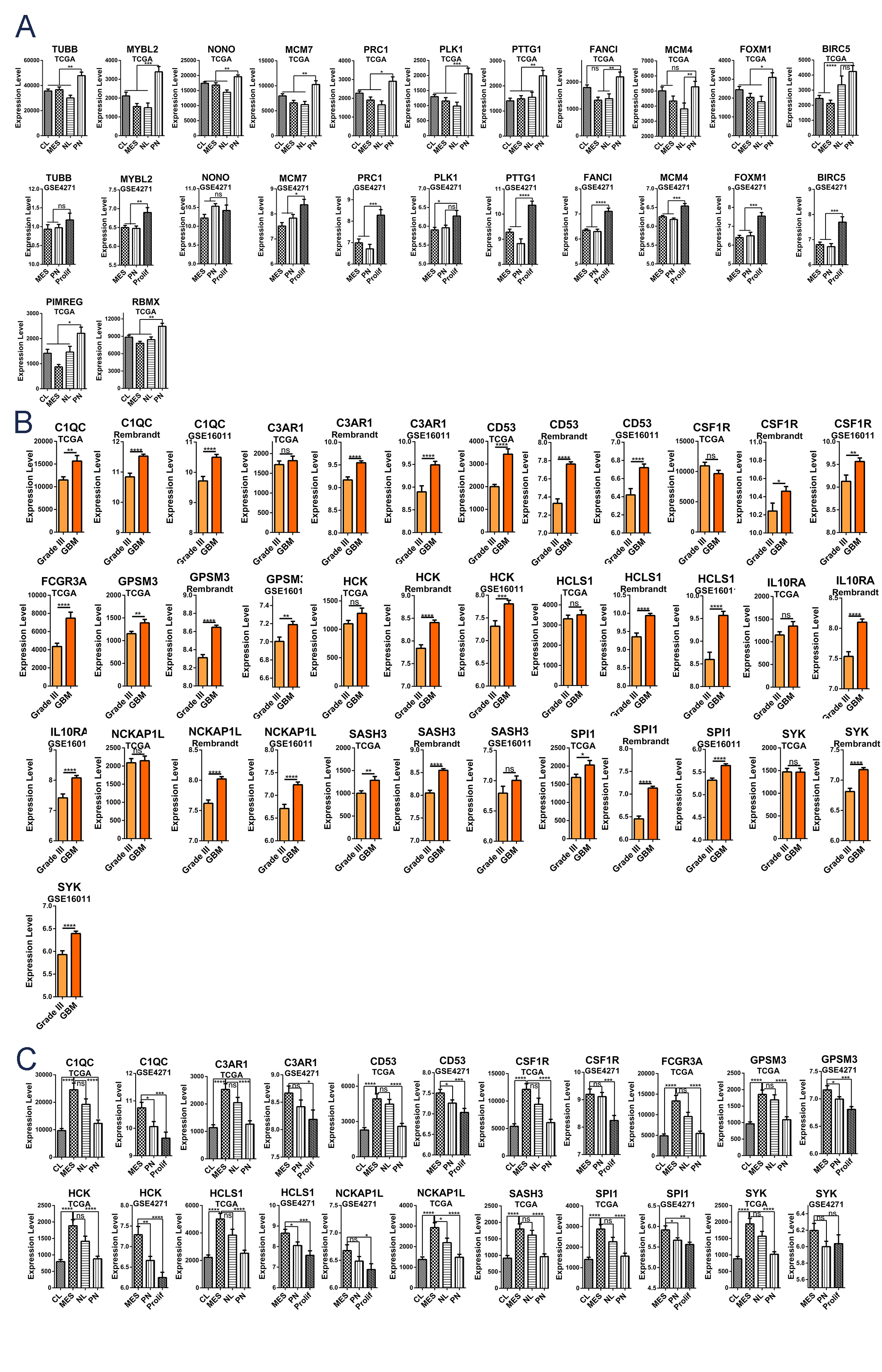

Supplement: Supplementary file 5 [file JCMM-24-3901-s005.tif]

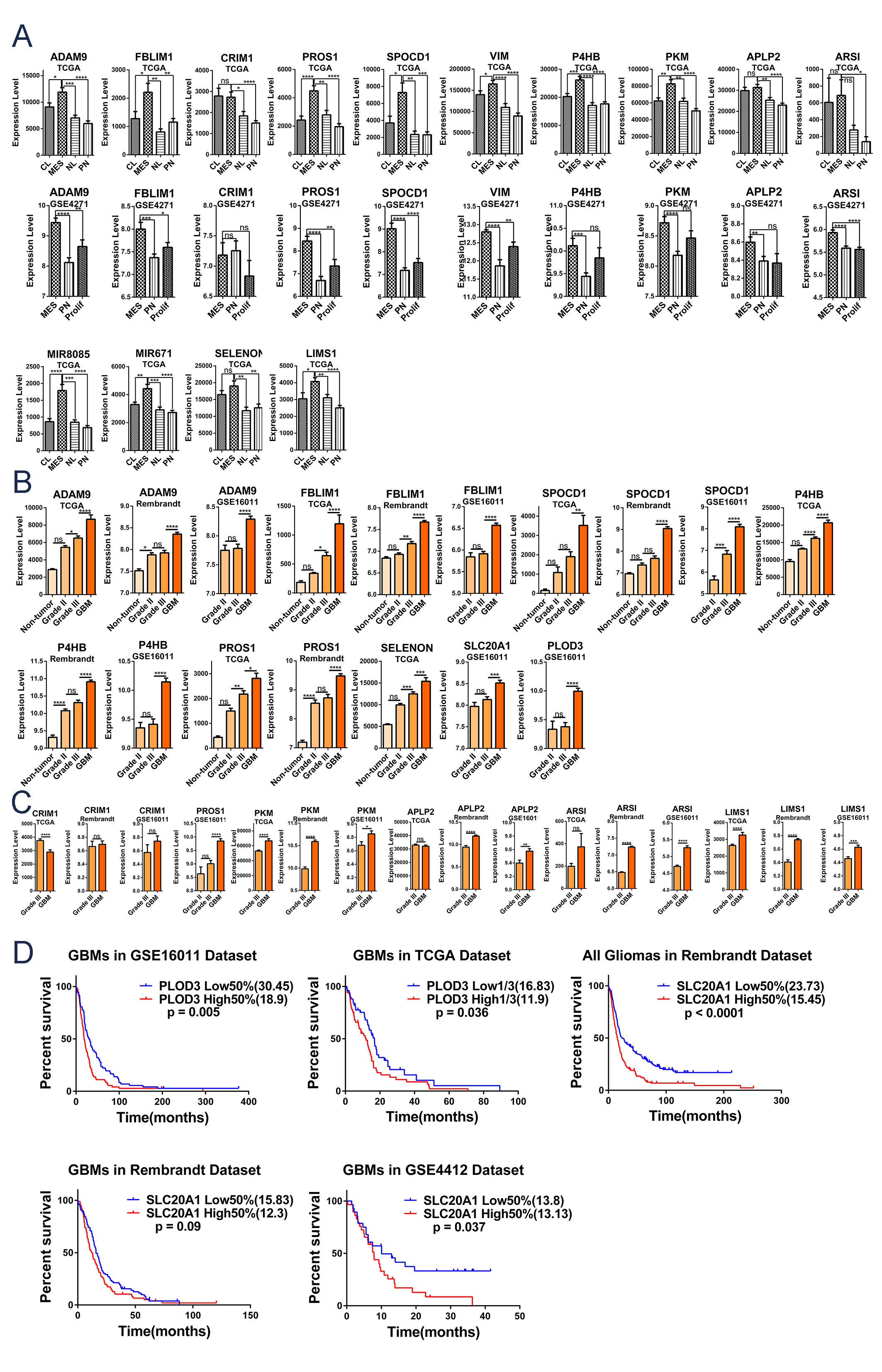

Supplement: Supplementary file 6 [file JCMM-24-3901-s006.tif]

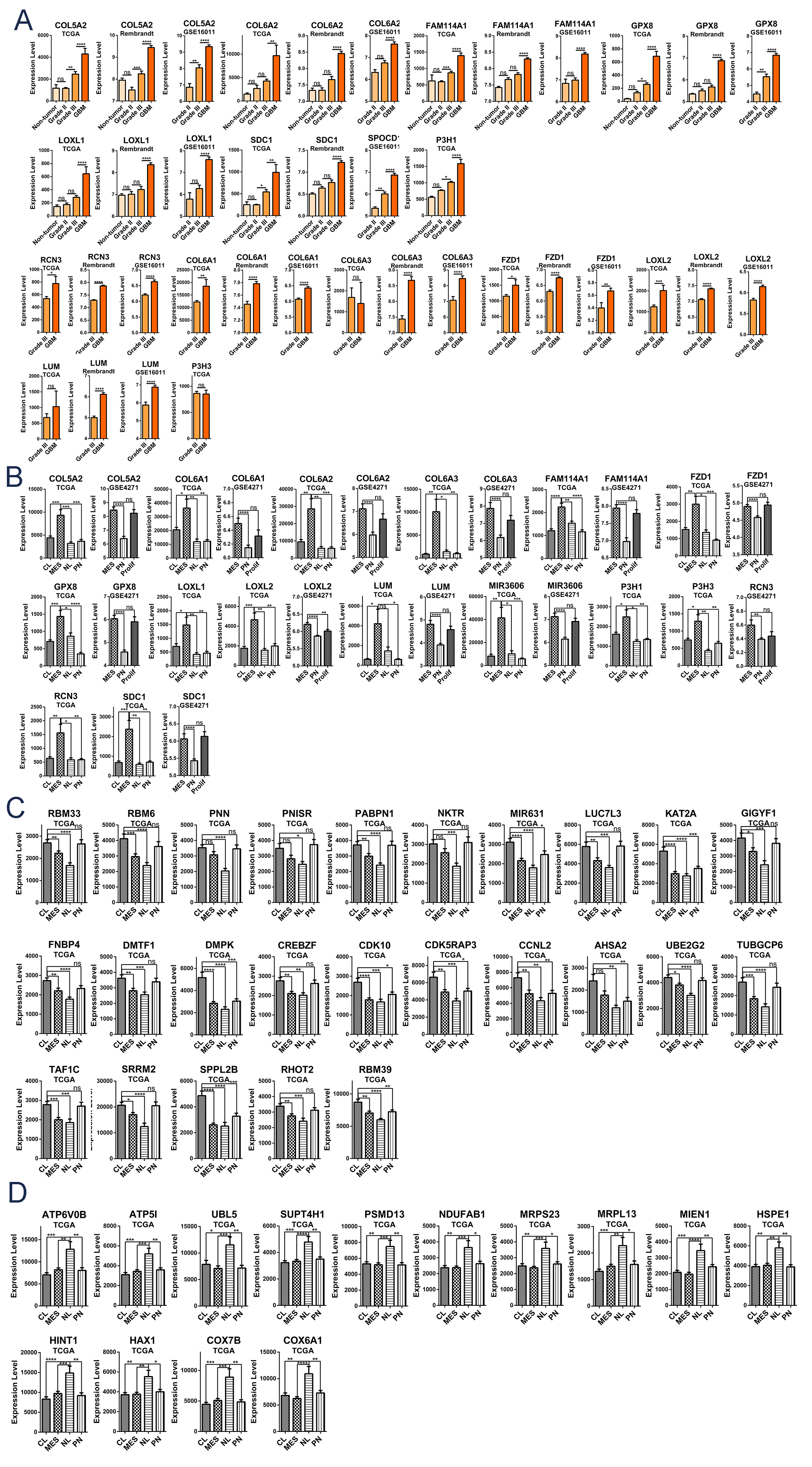

Supplement: Supplementary file 7 [file JCMM-24-3901-s007.tif]

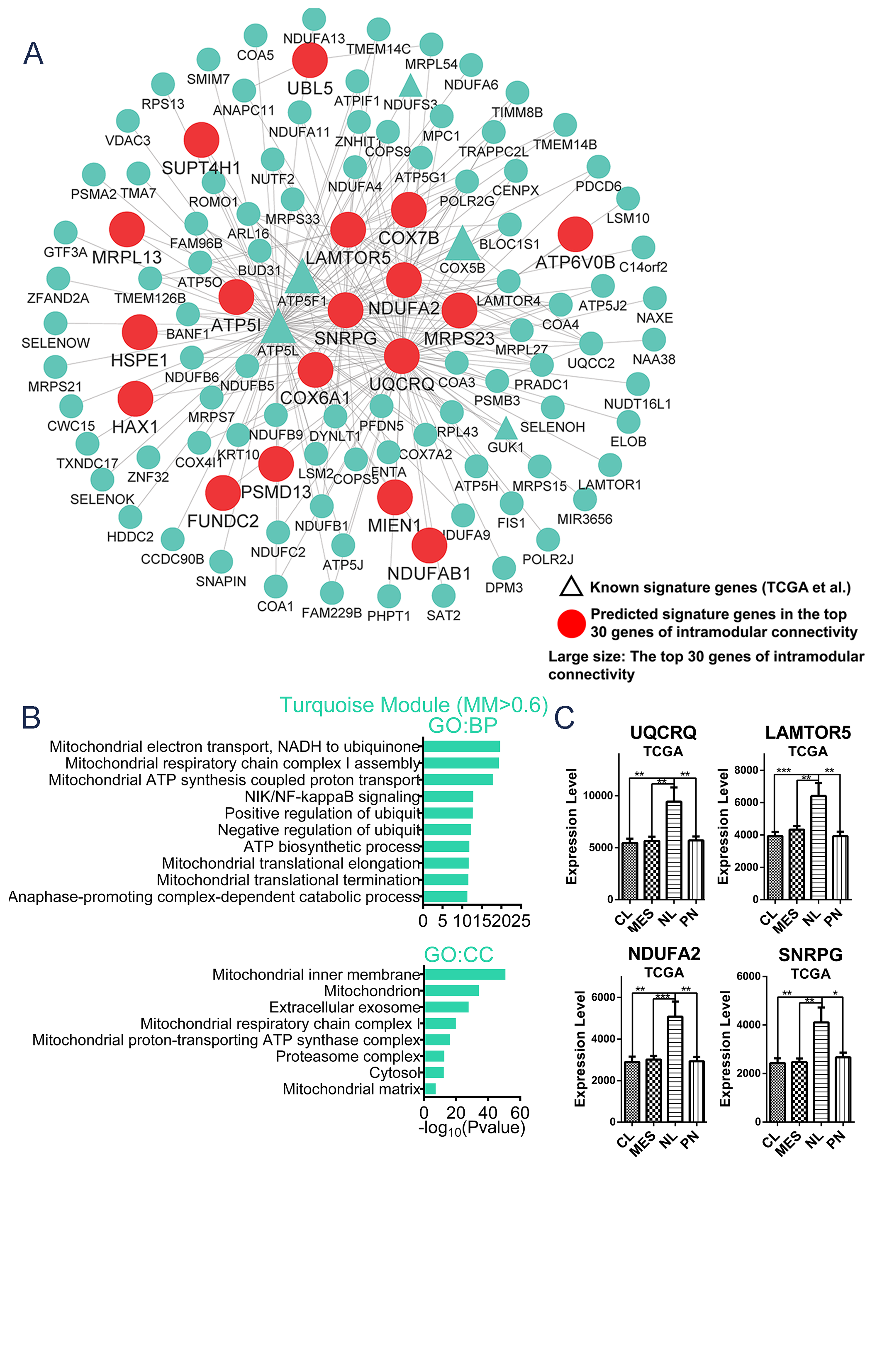

Supplement: Supplementary file 8 [file JCMM-24-3901-s008.tif]

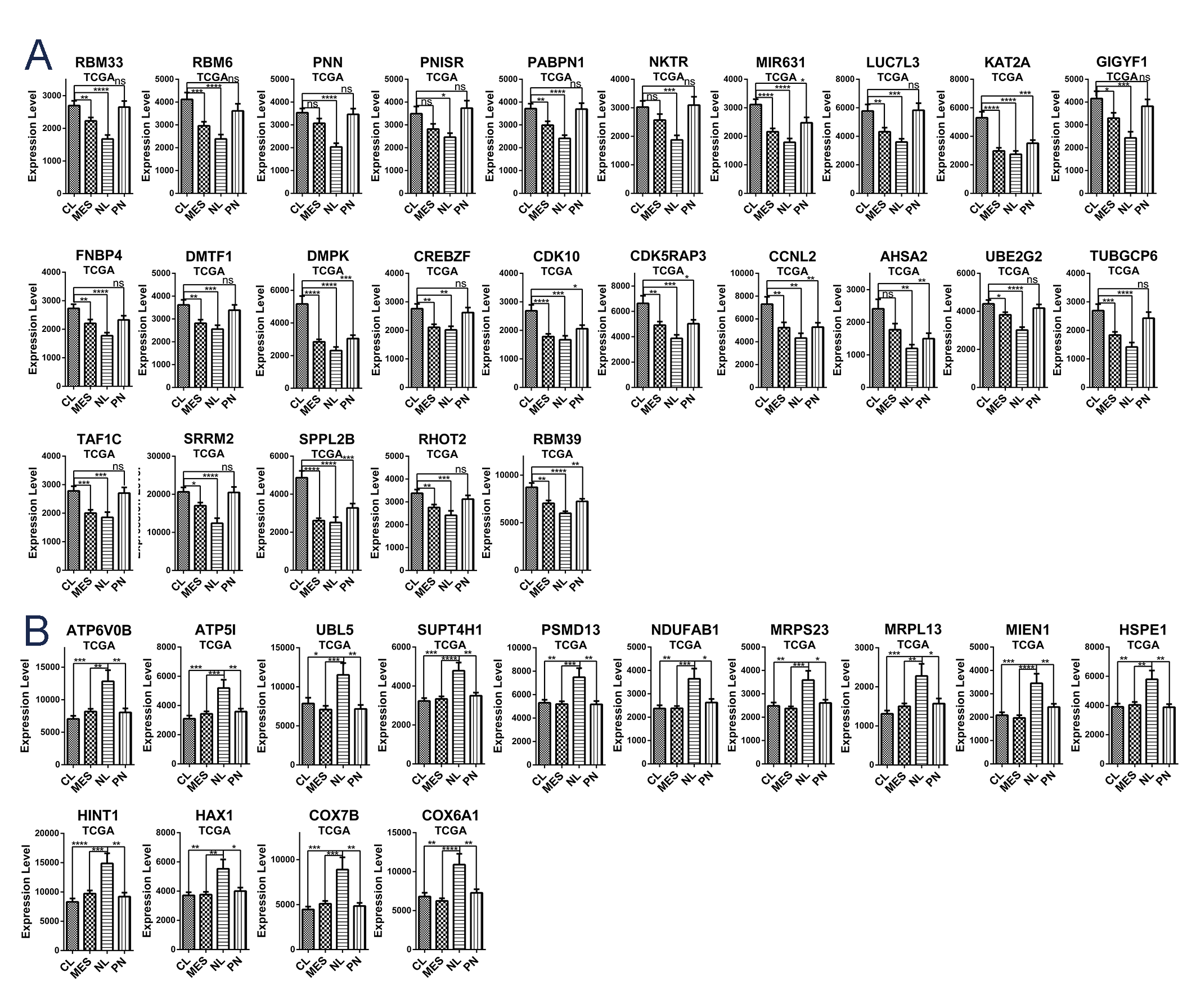

Supplement: Supplementary file 9 [file JCMM-24-3901-s009.tif]
