# Supplementary material for: Transcriptome analyses reveal molecular mechanisms underlying phenotypic differences among transcriptional subtypes of glioblastoma
Source: J Cell Mol Med. 2020 Feb 24;24(7):3901–16. doi: 10.1111/jcmm.14976 (PMC7171397; doi:10.1111/jcmm.14976)
Supplement: Supplementary file 10 [file JCMM-24-3901-s010.docx]

**Table S1. Immunohistochemistry results and clinical features of patients with different grade gliomas.**

| Protein | Patient ID | Sex | Age | Grade | Staining | Intensity | Quantity | Location | Antibody | | |
| --- | --- | --- | --- | --- | --- | --- | --- | --- | --- | --- | --- |
| PLOD3 | 158 | Female | 15 | LGG | Not detected | Negative | Negative | None | HPA001236 | | |
| PLOD3 | 34 | Female | 36 | LGG | Not detected | Negative | Negative | None | HPA001236 | | |
| PLOD3 | 221 | Female | 37 | HGG | Not detected | Negative | Negative | None | HPA001236 | | |
| PLOD3 | 206 | Male | 66 | HGG | High | Strong | >75% | Cytoplasmic/membranous | HPA001236 | | |
| PLOD3 | 38 | Male | 36 | HGG | Low | Weak | >75% | Cytoplasmic/membranous | HPA001236 | | |
| PLOD3 | 191 | Male | 69 | HGG | Medium | Moderate | >75% | Cytoplasmic/membranous | HPA001236 | | |
| PLOD3 | 45 | Male | 72 | HGG | Not detected | Negative | Negative | None | HPA001236 | | |
| PLOD3 | 183 | Male | 48 | HGG | Low | Weak | 75%-25% | Cytoplasmic/membranous | HPA001236 | | |
| PLOD3 | 105 | Female | 60 | HGG | Not detected | Weak | <25% | Cytoplasmic/membranous | HPA001236 | | |
| PLOD3 | 3 | Male | 68 | HGG | Not detected | Negative | Negative | None | HPA001236 | | |
| PLOD3 | 46 | Male | 77 | HGG | Medium | Moderate | >75% | Cytoplasmic/membranous | HPA001236 | | |
| PLOD3 | 223 | Female | 65 | HGG | Low | Weak | 75%-25% | Cytoplasmic/membranous | HPA001236 | | |
| SLC20A1 | 93 | Female | 58 | LGG | Medium | Moderate | >75% | Cytoplasmic/membranous | CAB019279 | | |
| SLC20A1 | 122 | Female | 32 | LGG | Medium | Moderate | >75% | Cytoplasmic/membranous | CAB019279 | | |
| SLC20A1 | 2909 | Male | 43 | LGG | Low | Weak | 75%-25% | Cytoplasmic/membranous | CAB019279 | | |
| SLC20A1 | 2874 | Male | 23 | HGG | Medium | Moderate | >75% | Cytoplasmic/membranous | CAB019279 | | |
| SLC20A1 | 2871 | Male | 55 | HGG | High | Strong | >75% | Cytoplasmic/membranous | CAB019279 | | |
| SLC20A1 | 2856 | Male | 53 | HGG | Low | Moderate | <25% | Cytoplasmic/membranous | CAB019279 | | |
| SLC20A1 | 2849 | Male | 54 | HGG | Medium | Moderate | 75%-25% | None | CAB019279 | |  |
| SLC20A1 | 2879 | Female | 65 | HGG | Medium | Moderate | >75% | Cytoplasmic/membranous | CAB019279 | |  |
| SLC20A1 | 2907 | Male | 76 | HGG | Medium | Moderate | >75% | Cytoplasmic/membranous | CAB019279 | |  |
| SLC20A1 | 2862 | Female | 40 | HGG | Medium | Moderate | >75% | Cytoplasmic/membranous | CAB019279 | |  |
| SLC20A1 | 2905 | Male | 68 | HGG | Not detected | Negative | Negative | None | CAB019279 | |  |
| CKAP4 | 176 | Female | 46 | LGG | High | Strong | >75% | Cytoplasmic/membranous | HPA000729 | |  |
| CKAP4 | 158 | Female | 15 | LGG | Medium | Strong | <25% | Cytoplasmic/membranous | HPA000729 | |  |
| CKAP4 | 34 | Female | 36 | LGG | High | Strong | 75%-25% | Cytoplasmic/membranous | HPA000729 | |  |
| CKAP4 | 221 | Female | 37 | HGG | High | Strong | 75%-25% | Cytoplasmic/membranous | HPA000729 | |  |
| CKAP4 | 206 | Male | 66 | HGG | Medium | Moderate | 75%-25% | Cytoplasmic/membranous | HPA000729 | |  |
| CKAP4 | 38 | Male | 36 | HGG | High | Strong | >75% | Cytoplasmic/membranous | HPA000729 | |  |
| CKAP4 | 191 | Male | 69 | HGG | High | Strong | >75% | Cytoplasmic/membranous | HPA000729 | |  |
| CKAP4 | 45 | Male | 72 | HGG | Low | Moderate | <25% | Cytoplasmic/membranous | HPA000729 | |  |
| CKAP4 | 183 | Male | 48 | HGG | High | Strong | >75% | Cytoplasmic/membranous | HPA000729 | |  |
| CKAP4 | 105 | Female | 60 | HGG | High | Strong | >75% | Cytoplasmic/membranous | HPA000729 | |  |
| CKAP4 | 3 | Male | 68 | HGG | High | Strong | 75%-25% | Cytoplasmic/membranous | HPA000729 | |  |
| CKAP4 | 46 | Male | 77 | HGG | Low | Weak | 75%-25% | Cytoplasmic/membranous | HPA000729 | |  |
| PCOLCE | 3137 | Male | 77 | LGG | Not detected | Negative | Negative | None | CAB017623 | |  |
| PCOLCE | 2529 | Female | 37 | LGG | Not detected | Negative | Negative | None | CAB017623 |  |  |
| PCOLCE | 3120 | Male | 38 | LGG | Not detected | Weak | <25% | Cytoplasmic/membranous | CAB017623 |  |  |
| PCOLCE | 3174 | Female | 22 | LGG | Not detected | Weak | <25% | Cytoplasmic/membranous | CAB017623 |  |  |
| PCOLCE | 1587 | Female | 36 | HGG | Not detected | Negative | Negative | None | CAB017623 |  |  |
| PCOLCE | 3151 | Male | 32 | HGG | Not detected | Negative | Negative | None | CAB017623 |  |  |
| PCOLCE | 2726 | Male | 60 | HGG | Not detected | Negative | Negative | None | CAB017623 |  |  |
| PCOLCE | 1627 | Male | 33 | HGG | Not detected | Negative | Negative | None | CAB017623 |  |  |
| PCOLCE | 3421 | Female | 58 | HGG | Medium | Moderate | 75%-25% | Cytoplasmic/membranous | CAB017623 |  |  |
| PCOLCE | 1645 | Female | 59 | HGG | Not detected | Negative | Negative | None | CAB017623 |  |  |
| PCOLCE | 3091 | Male | 71 | HGG | Medium | Moderate | 75%-25% | Cytoplasmic/membranous | CAB017623 |  |  |

LGG: Low grade glioma; HGG: High grade glioma
